# Single-cell sequencing uncovers clonal dynamics profiles and therapeutic resistance biomarkers in relapsed and refractory peripheral T-cell lymphoma

**DOI:** 10.3389/fimmu.2026.1790664

**Published:** 2026-07-10

**Authors:** Ning Zhang, Jingwei Yu, Hengqi Liu, Zechao Hu, Shen Meng, Cong Sun, Yaxiao Lu, Xia Liu, Zheng Song, Lanfang Li, Lihua Qiu, Zhengzi Qian, Bin Meng, Wenchen Gong, Shiyong Zhou, Xianhuo Wang, Huilai Zhang

**Affiliations:** 1 State Key Laboratory of Druggability Evaluation and Systematic Translational Medicine/Department of Lymphoma, Tianjin Medical University Cancer Institute and Hospital, National Clinical Research Center for Cancer, Tianjin’s Clinical Research Center for Cancer, Key Laboratory of Cancer Prevention and Therapy, the Sino-US Center for Lymphoma and Leukemia Research, Tianjin, China; 2 Department of Radiation Oncology, Shandong Cancer Hospital and Institute, Shandong First Medical University, Shandong Academy of Medical Sciences, Jinan, China; 3 Department of Pathology, Tianjin Medical University Cancer Institute and Hospital, Tianjin, China

**Keywords:** clonal dynamics, epigenetic regulation, relapsed and refractory peripheral T-cell lymphoma, single-cell sequencing, transcriptional features

## Abstract

**Introduction:**

Peripheral T-cell lymphoma (PTCL) is a heterogeneous and highly aggressive subtype of non-Hodgkin lymphoma. Approximately 30% of patients develop relapsed or refractory PTCL (R/R PTCL) due to disease recurrence or failure to achieve complete remission after first-line therapy. Despite therapeutic advances, the molecular and cellular mechanisms underlying treatment resistance in R/R PTCL remain unclear.

**Methods:**

Single-cell RNA sequencing and single-cell T-cell receptor sequencing were performed on seven tumor samples from six patients with R/R PTCL. These approaches were used to systematically characterize the transcriptional profiles of malignant T-cell clones and reactive T lymphocytes, define the transcriptomic landscape of R/R PTCL, and identify potential epigenetic biomarkers associated with drug response.

**Results:**

We observed significant upregulation of genes associated with cell proliferation, oncogenic signaling, and immune modulation in R/R PTCL. Within the tumor microenvironment, specific protumorigenic ligand–receptor interactions were identified, including CXCL13–CXCR5, CCL5–CCR5, and CD74–MIF interactions, which may facilitate immune evasion by malignant T cells. Longitudinal analysis of a patient who progressed following dual epigenetic therapy revealed marked downregulation of immune response-related genes, including HLA-DRA/DPA1/DRB5, CD74, C1QC, and LYZ, as well as functional reprogramming of tumor-associated macrophages. Enhanced LGALS9–HAVCR2 and CSF1–CSF1R interactions were also observed following combination treatment with chidamide and azacitidine.

**Discussion:**

This study delineates the transcriptional heterogeneity of malignant T-cell clones in R/R PTCL and suggests that this heterogeneity may contribute to resistance to epigenetic therapies. These findings provide novel insights into the molecular mechanisms underlying treatment resistance and highlight potential avenues for therapeutic intervention in R/R PTCL.

## Introduction

Peripheral T-cell lymphoma (PTCL) constitutes a rare and highly heterogeneous group of malignancies derived from mature T cells and natural killer (NK) cells (1). According to the latest World Health Organization classification of hematopoietic and lymphoid tumors, PTCL encompasses 34 distinct subtypes, including peripheral T-cell lymphoma–not otherwise specified (PTCL-NOS) and angioimmunoblastic T-cell lymphoma (AITL) (2). Despite advances in classification, the standard first-line therapy for aggressive PTCL subtypes remains the CHOP regimen (cyclophosphamide, doxorubicin, vincristine, and prednisone), which is broadly applied across subtypes (3, 4). However, clinical outcomes remain poor: the 5-year progression-free survival rate for PTCL patients, excluding those with ALK-negative anaplastic large cell lymphoma, is approximately 20%, and up to 30% of patients eventually develop relapsed or refractory disease (R/R PTCL) (5, 6). The molecular and cellular mechanisms underlying treatment resistance in patients with R/R PTCL remain largely elusive, despite advances in therapeutic strategies.

The pathogenesis of PTCL is highly complex and multifactorial and involves dysregulation of the T-cell receptor (TCR) signaling pathway, malignant transformation driven by viral infection and chronic inflammation, and widespread epigenetic alterations. Notably, epigenetic dysregulation has been increasingly recognized as a central mechanism underlying chemotherapy resistance and disease progression in patients with PTCL (7). The VALENTINE-PTCL 01 study demonstrated that valemetostat, an EZH1/2 inhibitor, achieved clinically meaningful and durable responses in patients with R/R PTCL across diverse histological subtypes (8). Preclinical studies have consistently shown that combining histone deacetylase inhibitors (HDACis) with DNA methyltransferase inhibitors (DNMTis) leads to the synergistic suppression of tumor growth in both *in vitro* and *in vivo* models of T-cell lymphoma (TCL) (9). These findings have been translated into clinical practice, as evidenced by phase I and II trials demonstrating the robust therapeutic efficacy of the azacitidine–romidepsin combination in both treatment-naïve and R/R PTCL patients (10, 11). Collectively, these findings strongly suggest that R/R PTCL exhibits distinct biological vulnerability to epigenetic therapies, highlighting the potential of targeting the epigenome as a cornerstone strategy in the management of this aggressive disease.

In this study, single-cell RNA sequencing (scRNA-seq) and single-cell T-cell receptor sequencing (scTCR-seq) were performed on seven tumor biopsy samples from six enrolled patients to define dominant malignant T-cell clones at single-cell resolution. We characterized the dominant malignant clonal profiles in each sample, revealing marked heterogeneity among R/R PTCL tumor cells. Transcriptomic analysis enabled the identification of cell type-specific gene expression patterns in shared clonal populations and revealed the potential therapeutic resistance biomarkers in R/R PTCL following treatment with dual epigenetic regulators.

## Patients and methods

### Patient samples and cell preparation

This study enrolled a total of six R/R PTCL patients from the Lymphoma Department of Tianjin Medical University Cancer Institute and Hospital. The pathological diagnosis of all enrolled patients was reconfirmed by pathologists. Among the six patients, four were diagnosed with PTCL-NOS (S_TCL001T, S_TCL002T, S_TCL006T and S_TCL008T), while the remaining two were diagnosed with AITL(S_TCL004T, S_TCL009T). Notably, paired longitudinal samples were collected from one PTCL-NOS patient before treatment initiation (S_TCL002T) and after disease progression (S_TCL003T) following treatment with chidamide combined with azacitidine (**Supplementary Table 1**). Based on whether the patients had received dual epigenetic regulator therapy at the time of puncture biopsy and their post-treatment disease status, the 7 samples were categorized into the pre-treatment group and the progressive disease group. Specifically, the pre-treatment group comprised six samples, while the progressive disease group included one sample. The study was conducted in accordance with the Declaration of Helsinki and approved by the institutional review boards of the Tianjin Medical University Cancer Institute and Hospital (Approval ID: E2020530). Informed consent was obtained from all patients.

The fresh tissues harvested from the patient’s puncture biopsy were washed with Hanks’ balanced salt solution, minced into small pieces, and then digested with sCelLive™ Tissue Dissociation Solution (Singleron, China) using a Singleron PythoN™ Tissue Dissociation System at 37 °C for 15 min. The cell suspension was collected and filtered through a sterile strainer. Afterward, GEXSCOPE^®^ red blood cell lysis buffer (Singleron) was added to remove red blood cells. The mixture was then centrifuged to remove the supernatant, after which the cells were gently suspended in PBS. Finally, quality control was conducted on the obtained single-cell suspensions.

### Droplet-based scRNA-seq

In accordance with the protocols of the Chromium single-cell 3’ and 5’ libraries, Gel Bead & Multiplex Kit (10x Genomics), single-cell suspensions at a concentration of 1×10^6^ cells per milliliter derived from lymph node puncture samples of six patients were transformed into barcoded single-cell RNA sequencing libraries. Briefly, the cells were partitioned into Gel Beads in Emulsion in the Chromium™ Controller instrument, where cell lysis and barcoded reverse transcription of RNA occurred. TCR- and BCR-enriched libraries were generated with aliquots from each of the aforementioned cDNAs using the Chromium Single-Cell V(D) J Enrichment Kit. All the libraries were sequenced on the Illumina NovaSeq 6000 platform with 150 bp paired-end reads.

### scRNA-seq data preprocessing

Droplet-based sequencing data were subjected to quality control using FastQC software. Afterward, reads were aligned to the GRCh38 human reference genome provided by Cell Ranger (version 2.0, 10× Genomics), and unique molecular identifier (UMI) counts were summarized for each gene in each cell. Functions from Seurat v3.1.2 (12) were used for dimensionality reduction and clustering. The NormalizeData and ScaleData functions were used to normalize and scale gene expression, and the top 2,000 highly variable genes were selected using the FindVariableFeatures function for principal component analysis (PCA). Based on the top 20 principal components, cells were separated into multiple clusters using FindClusters. Batch effects between samples were corrected using Harmony (13). Finally, Uniform Manifold Approximation and Projection (UMAP) was applied to visualize cells in a two-dimensional space.

### Cell type annotation

The cell types in each cluster were determined according to the expression of canonical markers from the reference database SynEcoSys™ (Singleron). SynEcoSys™ contains collections of canonical cell type markers for single-cell sequencing data from CellMakerDB, PanglaoDB and recently published literature (12, 14). During cell type annotation, potential doublet-enriched, ambiguous, or low-quality clusters were identified based on the aberrant co-expression of canonical markers from distinct cell lineages. Clusters harboring multiple lineage-specific marker signatures were excluded from downstream analyses to ensure the reliability of cell type annotation and subsequent biological interpretation. After major cell types were annotated and ambiguous clusters were excluded, T cells were extracted for further subclustering. T-cell re-clustering was performed using the Louvain algorithm implemented in the Seurat FindClusters function, with the resolution parameter set to 0.1. The resulting T-cell clusters were visualized using UMAP.

### Analysis of differentially expressed genes

We used the Seurat FindMarkers function based on the Wilcoxon likelihood ratio test with the default parameters to identify DEGs and selected genes expressed in more than 10% of the cells in a cluster and whose average log (fold change) value > 0.25 as DEGs. For the cell type annotation of each cluster, we combined the expression of canonical markers found in the DEGs with knowledge from the literature and displayed the expression of markers of each cell type in heatmaps/dot plots/violin plots that were generated with the Seurat DoHeatmap/DotPlot/Vlnplot function (15–17).

### TCR analyses

The V(D)J sequencing data were analyzed using Cell Ranger (version 3.1.0, https://github.com/10XGenomics/cellranger). After removing cells with only a single chain (light or heavy chain for BCR and α chain or β chain for TCR), the remaining cells were annotated to V(D)J data using the 5’ single-cell sequencing data.

### Pathway enrichment analyses

Gene Ontology (GO) and Kyoto Encyclopedia of Genes and Genomes (KEGG) analyses were performed with the “clusterProfiler” R package 3.16.1 to investigate the potential functions of the DEGs (18). Pathways with P value < 0.05 were considered significantly enriched. For the GSVA pathway enrichment analysis, the average gene expression in each cell type was used as input data and analyzed using the GSVA package (19).

### Trajectory analysis

The cell differentiation trajectory was reconstructed with Monocle 2 (20). Highly variable genes were used to sort cells in order of spatiotemporal differentiation. We used DDRTree to perform FindVairableFeatures and dimension reduction. Finally, the trajectory was visualized using the plot_cell_trajectory function.

### CellPhoneDB interaction analysis

Cell–cell interactions were analyzed using CellPhoneDB v2.1.0 based on known receptor–ligand interactions between two cell types/subtypes (21). The cluster labels of all the cells were randomly permuted 1000 times to calculate the null distribution of the average ligand–receptor expression levels of the interacting clusters. Individual ligand or receptor expression was thresholded with a cutoff value based on the average log gene expression distribution for all genes across all the cell types. Significant cell–cell interactions were defined as a P value < 0.05 and an average log expression > 0.1, which were visualized with the dot plot v0.4.10 R package.

## Results

### Single-cell analysis revealed the transcriptomic landscape of R/R PTCL

**Figure 1:**
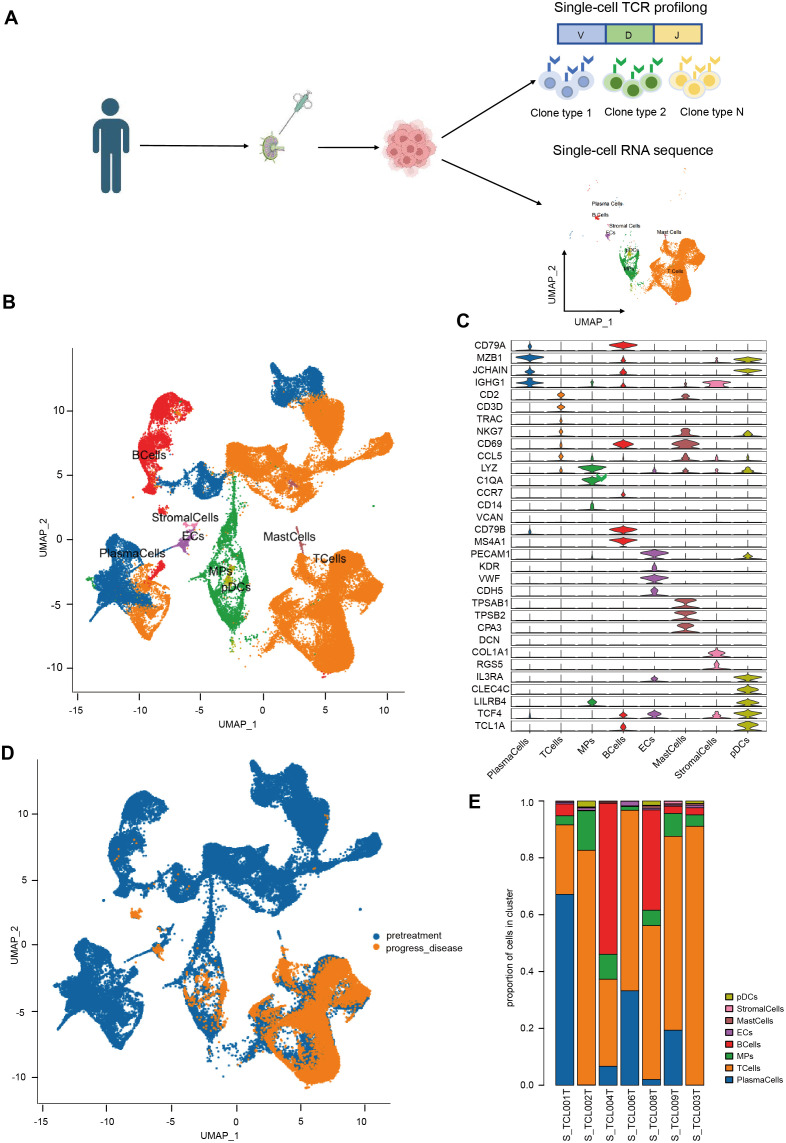
Single-cell transcriptome map of R/R PTCL. **(A)** Sample acquisition and workflow. **(B)** UMAP visualization shows the expression profiles of 70009 cells from 7 samples that passed quality control (each dot represents one cell, and different colors represent different cell types). **(C)** Violin diagram of typical cell type markers by cell type. Each color corresponds to a distinct cell type. **(D)** UMAP visualizations of the two cell groups are presented separately for the pre- and postepigenetic drug treatment states, with the pretreatment group comprising six samples: S_TCL001T, S_TCL002T, S_TCL004T, S_TCL006T, S_TCL008T, and S_TCL009T. The progressive disease group consists of one sample, S_TCL003T. Different colors are employed to denote different groups. **(E)** Histogram showing the proportion of each cellular subpopulation in each sample.

We performed scRNA-seq and scTCR-seq on seven core needle biopsy samples from six patients to investigate the heterogeneity of the (TME) in R/R PTCL (**Figure 1A**). Based on whether patients had received dual epigenetic regulatory therapy at the time of biopsy and their disease status after treatment, the samples were stratified into a pretreatment group and a progressive disease group. Raw single-cell RNA sequencing data were subjected to rigorous quality control (see the Methods; **Supplementary Table 2**). A total of 70,009 high-quality cells from seven samples were retained and visualized in a UMAP plot. Using canonical cell type-specific markers and transcriptional profiles, eight distinct cell populations were identified: T cells, B cells, plasma cells, mononuclear phagocytes, plasmacytoid dendritic cells (pDCs), mast cells, stromal cells, and endothelial cells (ECs) (**Figures 1B, C**; **Supplementary Table 3**). Cells clustered predominantly by lineage, with stromal and immune cells grouped according to their respective types across samples, reflecting both conserved cellular identities and pronounced tumor heterogeneity (**Figure 1D**; **Supplementary Figures 1A–C**). The relative abundance of each cellular subpopulation varied considerably across the seven samples (**Figure 1E**; **Supplementary Figure 1D**). These findings indicate that the TME in R/R PTCL functions as a multicellular ecosystem composed of diverse cellular components.

### Dominant malignant clonal expansion of heterogeneous T cells across R/R PTCL samples

**Figure 2:**
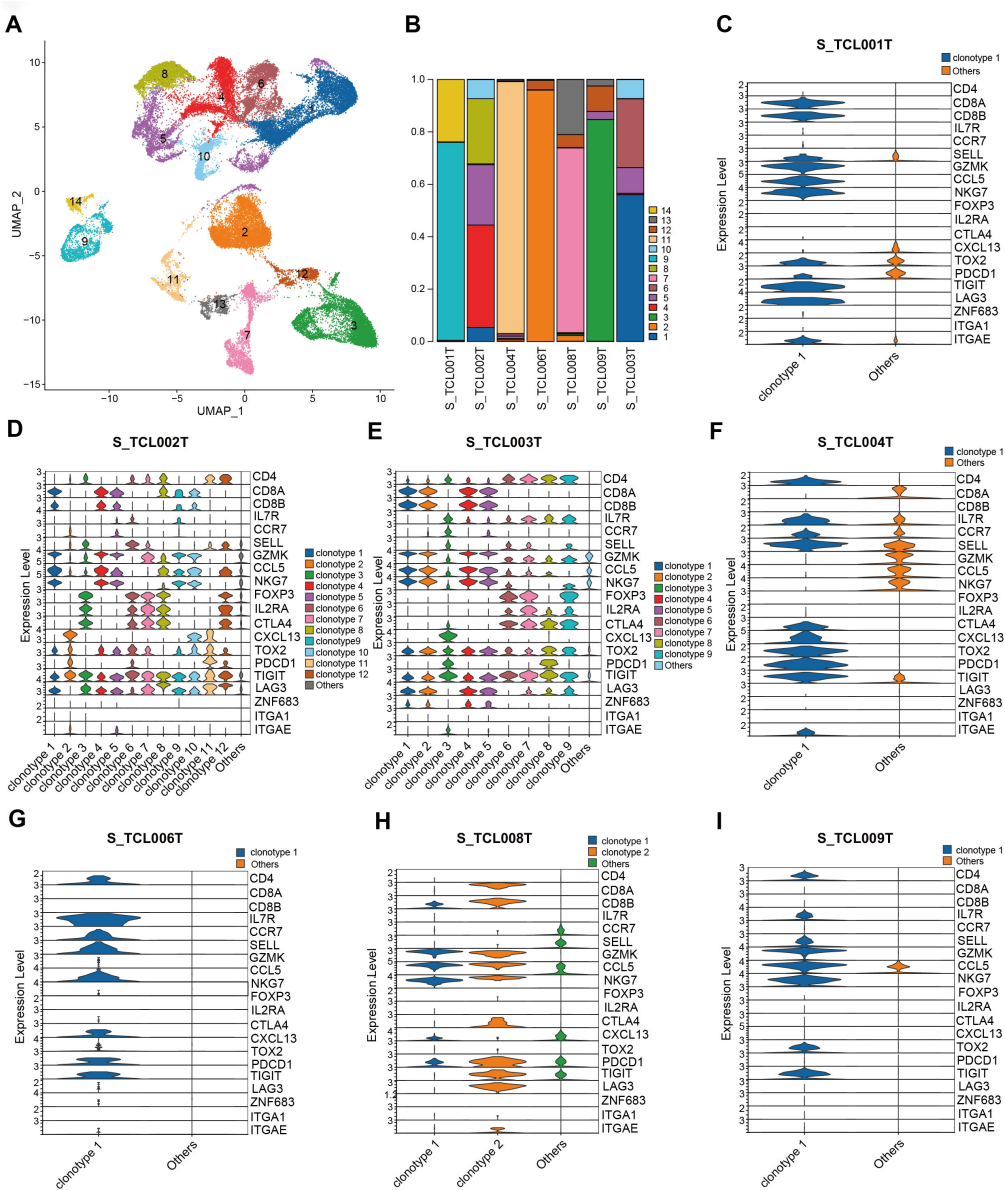
Expansion of dominant malignant clonotypes among heterogeneous T cells in R/R PTCL samples. **(A)** UMAP plot of T-cell clusters (each dot represents a cell, and different colors represent different cell subsets). **(B)** Histogram showing the proportion of each cellular subpopulation in each sample. **(C-I)** Violin plot illustrating the average expression levels of signature genes of dominant clonal T cells relative to those of nonclonal T cells derived from the same tumor (proportionally scaled). Detailed information on the methodology for assessing differentially expressed genes is provided in the Materials and Methods section.

To further characterize the heterogeneity of the T-cell compartment in R/R PTCL, annotated T cells were extracted and subjected to re-clustering, which revealed 14 T-cell clusters with distinct transcriptional profiles and marked variation in cluster composition across samples (**Figures 2A, B**). Based on the scTCR-seq clonotype frequency distribution and the pathological context of our PTCL cohort, clonotypes comprising ≥40 cells were designated as dominant malignant clonotypes, whereas those comprising <40 cells were grouped into the “Others” category. These clonotypes were mapped onto the UMAP space for each sample, and the top five differentially expressed genes for each dominant malignant clonotype and the “Others” group were visualized to characterize their transcriptional identities (**Supplementary Table 4**). Dominant malignant clonal types were identified in all the samples, with variable proportions relative to the total T-cell population across the samples (**Figures 2C–I**; **Supplementary Table 5**). We integrated the expression profiles of canonical cell type markers with transcriptomic data to characterize the cellular origins of T cells within each dominant clonotype (**Supplementary Table 6**). Our analysis revealed distinct but overlapping lineage signatures across samples (**Supplementary Figure 2**). Importantly, while each dominant malignant clonotype predominantly expressed markers defining a specific T-cell subset, low-to-moderate expression of markers associated with alternative lineages was consistently observed. This result suggests that the T cells within individual clonotypes are not strictly homogeneous but may exhibit transcriptional features of multiple functional subsets, indicating potential phenotypic plasticity or heterogeneity within expanded clones.

### Transcriptional characteristics of dominant malignant T cell clones in R/R PTCL samples

**Figure 3:**
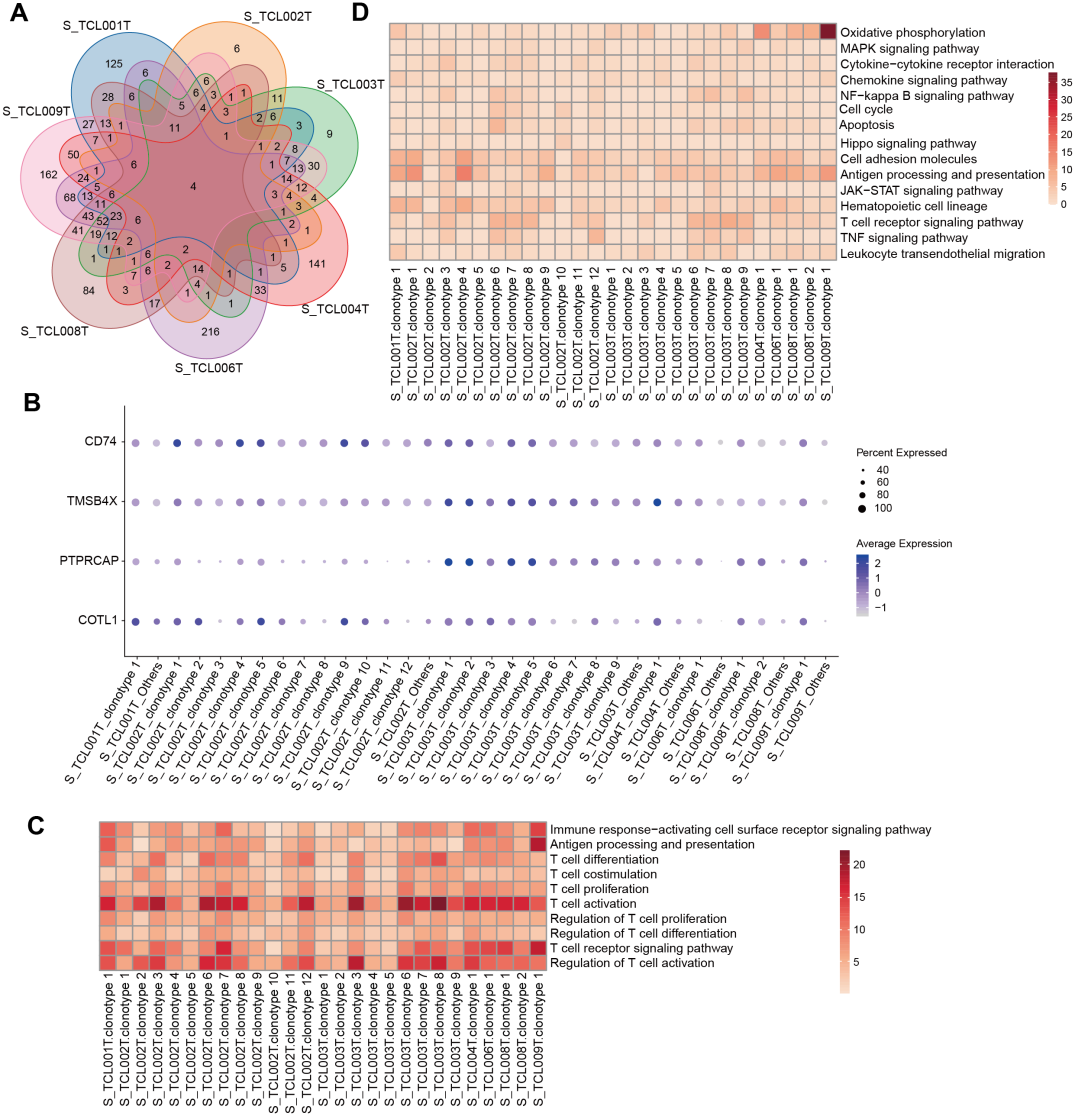
The global transcriptional signatures of dominant clonal T cells in R/R PTCL. **(A)** Venn diagram depicting the genes whose expression was globally upregulated in the dominant clonal T-cell populations across all the samples. **(B)** The expression profiles of globally upregulated genes in the dominant clonal T-cell populations across individual dominant clonal T cells in each sample (proportionally scaled). **(C, D)** Differentially expressed genes were identified using a significance threshold of P < 0.05. GO **(C)** and KEGG **(D)** enrichment analyses were subsequently conducted to characterize the functional pathways associated with the genes that were differentially expressed between the dominant and nondominant T cells in each sample.

We constructed a Venn diagram of upregulated genes across dominant clonotype populations to define the transcriptional profiles of the dominant clonotypes in each sample. This integrative approach enabled the identification of shared and sample-specific transcriptional signatures. In the pretreatment group, six samples exhibited a core set of six commonly upregulated genes within their dominant clonotypes: HLA-DRB1, HLA-DPB1, HLA-DRB5, HLA-DQB1, HLA-DQA1, and HLA-DPA1. In S_TCL002T and S_TCL003, 11 genes were coupregulated in the dominant clonotypes. A set of four genes—CD74, TMSB4X, PTPRCAP, and COTL1—was consistently upregulated across the seven samples (**Figure 3A**). Further examination revealed that TMSB4X and PTPRCAP were preferentially expressed in the dominant clonotype of S_TCL003T. In contrast, CD74 and COTL1 were expressed at higher levels in the dominant clonotypes of S_TCL001T and S_TCL002T within the pretreatment cohort (**Figure 3B**), indicating differential gene expression patterns among clonally expanded populations.

We performed GO and KEGG pathway enrichment analyses of the differentially expressed genes identified by comparing each dominant clonotype with all the other T cells from the same sample. Our results revealed the significant enrichment of pathways associated with oncogenic processes in dominant clonotypes. GO analysis of the upregulated genes revealed that these genes were predominantly involved in key biological processes, including T-cell proliferation, activation, differentiation, TCR signaling, and immune response-mediated activation of cell surface receptor signaling pathways (**Figure 3C**). According to the results of the KEGG pathway analysis (**Figure 3D**), in the pretreatment group, the dominant clones across the six samples were predominantly enriched in the “antigen processing and presentation” pathway. Notably, in S_TCL003, a sample from a patient who progressed following epigenetic drug treatment, the dominant clone was enriched primarily in the “TCR signaling pathway”.

### Functional landscape of dominant T malignant cell clones in pretreatment specimens

We performed ssGSVA across 50 HALLMARK gene sets using the entire population of such cells from each of the six samples in the pretreatment group. The results indicated that across these six samples, the upregulated differentially expressed genes were predominantly enriched in gene sets associated with cell proliferation, such as MYC_TARGETS_V1, MYC_TARGETS_V2, E2F_TARGETS, and P53_PATHWAY; in gene sets related to carcinogenesis, including DNA_REPAIR, PI3K_AKT_MTOR_SIGNALING, and OXIDATIVE_PHOSPHORYLATION; and in immune-related gene sets, namely, INTERFERON_ ALPHA/GAMMA_RESPONSE (**Supplementary Figure 3**). Furthermore, we performed a comprehensive analysis of cell–cell interactions among dominant clonal T cells, macrophages, and B cells in six samples from the pretreatment cohort. Our results revealed that, within immune checkpoint-related interaction pairs, dominant clonal T cells communicate with macrophages and B cells through multiple ligand–receptor axes. Notably, the LGALS9–HAVCR2 and TIGIT–NECTIN2 receptor–ligand pairs exhibited strong intercellular interactions between macrophages and dominant clonal T cells across all the samples (**Supplementary Figure 4**). With respect to chemokine-related receptor–ligand pairs, CXCL13–CXCR5, CCL5–CCR5, and CCL5–CCR1 showed robust interactions both between B cells and dominant clonal T cells and between macrophages and dominant clonal T cells (**Supplementary Figure 5**). In terms of cytokine-related interactions, the CD74–MIF axis displayed significant cross-talk between B cells and dominant clonal T cells as well as between macrophages and dominant clonal T cells (**Supplementary Figure 6**). Collectively, these cell–cell interaction data indicate that the consistent upregulation of LGALS9–HAVCR2 and TIGIT–NECTIN2 suggests an immunosuppressive TME. In parallel, elevated expression of CXCL13–CXCR5, CCL5–CCR5, and CD74–MIF may promote tumor immune evasion, thereby contributing to increased tumor cell proliferation, migration, and invasion.

### Clonal dynamics of the dominant T cell clonotypes in a patient before and after therapeutic intervention

**Figure 4:**
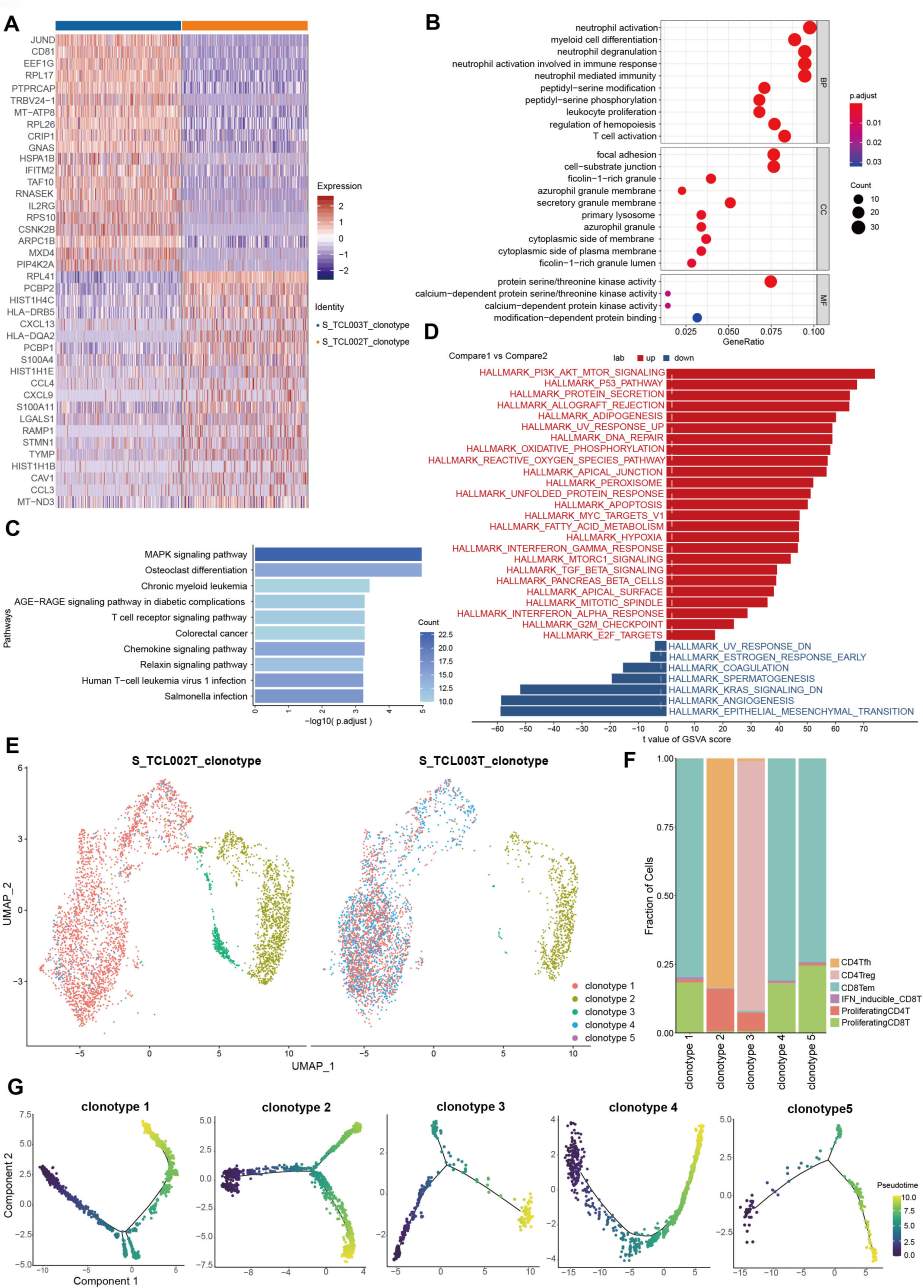
Transcriptomic differences in dominant malignant clonotypes between paired samples from the same patient before and after dual epigenetic therapy. **(A)** Heatmap displaying the global expression profile of differentially expressed genes in dominant clonal T cells from paired samples from the same patient before and after epigenetic drug treatment. Upregulated genes are shown in red, and downregulated genes are shown in blue. S_TCL002T represents a pretreatment sample from a patient exposed to dual epigenetic drugs, while S_TCL003T denotes a posttreatment progressive sample obtained following the administration of dual epigenetic drugs. **(B, C)** GO **(B)** and KEGG **(C)** enrichment analyses were performed on genes whose expression was upregulated in dominant clonal T cells in paired samples from the same patient before and after treatment. **(D)** GSVA was performed to evaluate the enrichment of 50 HALLMARK gene sets among the DEGs of dominant clonal T cells in paired pre- and posttreatment samples from the same patient. **(E)** UMAP visualization of the common dominant clonal T cells in two samples. **(F)** Histogram showing the relative proportions of functional T cell subsets in paired samples before and after treatment. **(G)** The single-cell analysis of the pseudotime trajectory of the same dominant clonal-type T cells from the same patient before and after treatment was performed using Monocle 2. A continuous value from 0 to 10 was assigned to each cell as a pseudotime.

The combination of chidamide and azacitidine in the treatment of PTCL enables the concurrent modulation of histone acetylation and DNA methylation, thereby suppressing tumor cell proliferation through dual epigenetic mechanisms and increasing therapeutic efficacy (22, 23). Samples S_TCL002T and S_TCL003T were obtained from the same patient prior to treatment with chidamide in combination with azacitidine and at the time of disease progression following therapy. We performed a differential gene expression analysis on the dominant clonal cell populations between the two samples. The differentially expressed genes were subsequently subjected to functional enrichment analyses using GO, KEGG, and a curated set of 50 HALLMARK pathways. Compared with the pretreatment sample S_TCL002T, the posttreatment sample S_TCL003T exhibited the significant downregulation of genes encoding histone H1 family members (HIST1H4C, HIST1H1E, and HIST1H1B), chemokines (CXCL13, CCL4, CXCL9, and CCL3), and major histocompatibility complex class II (MHC-II) molecules (HLA-DRB5 and HLA-DQA2) within the dominant clonal T-cell population. In contrast, genes such as JUND, CD81, EEF1G, and IFITM2 were significantly upregulated (**Figure 4A**). GO functional enrichment analysis revealed that the upregulated genes were predominantly enriched in biological processes related to the regulation of cell proliferation and immune responses (**Figure 4B**). KEGG pathway analysis revealed significant enrichment in the MAPK signaling pathway, TCR signaling pathway, and chemokine signaling pathway (**Figure 4C**). GSVA-based enrichment across the 50 HALLMARK gene sets showed strong activation of pathways such as PI3K_AKT_MTOR_SIGNALING, P53_PATHWAY and TGF_BETA_SIGNALING (**Figure 4D**). The integrated analysis of pre- and posttreatment differential expression profiles suggested that the genes HIST1H4C, HIST1H1E, HIST1H1B, CXCL13, HLA-DRB5, and HLA-DQA2 may be associated with the therapeutic response to the combination regimen of the epigenetic modulator chidamide and injectable azacitidine.

### Clonal dynamics of shared clonotypes in paired samples from the same patient before and after dual epigenetic therapy

A comparative analysis of TCR clonotypes between samples S_TCL002T and S_TCL003T revealed five shared clonotypes. A systematic evaluation of these identical clonotypes (**Supplementary Table 7**) showed that the frequencies of clonotype 1, 2, and 3 were lower in posttreatment samples than in pretreatment samples. Notably, clonotype 3 shifted from a dominant status to a nondominant status following treatment. Although the frequencies of clonotype 1 and 2 decreased after therapy, they remained among the predominant clonotypes in the posttreatment phase. In contrast, the frequencies of clonotype 4 and 5 increased after treatment (**Figure 4E**). We further investigated the functional dynamics of T cells within dominant clonal populations before and after treatment. Unsupervised clustering analysis based on the gene expression profiles of functional T cell signature genes revealed distinct functional T cell subsets among the dominant clonal T cells (**Supplementary Figure 7**). Notably, CD4^+^ regulatory T (Treg) cells emerged as the predominant population specifically enriched in clonotype 3 subset (**Figure 4F**). We investigated these results by performing a differential gene expression analysis on the same clonotypes within the same patient before and after treatment. The comparative assessment of the five shared clonotypes revealed largely consistent transcriptional changes in clonotype 1, 2, 4, and 5 across time points. However, clonotype 3 displayed the significant upregulation of genes involved in cell proliferation (JUND, NME2, HSPA1B, and PMAIP1) and immune regulation (PTPRCAP, IL2RG, MBD2, and CD81) following treatment (**Supplementary Figure 8**). We applied Monocle 2 to perform a pseudotime trajectory analysis of clonal expansion in identical T-cell clonotypes during T-cell differentiation before and after dual epigenetic therapy. Based on the transcriptomic profiles, we conducted a clustering analysis and mapped all T cells from the same clonotypes onto inferred developmental trajectories (**Figure 4G**). Cells were ordered along the default pseudotime axis, revealing that, except for clonotype 3, pre-treatment cells were primarily localized at earlier stages, whereas posttreatment cells predominantly occupied later stages (**Supplementary Figure 9A**). Dynamic gene expression analysis across pseudotime revealed that, in clonotype 1, clonotype 2, clonotype 4, and clonotype 5, genes associated with histone gene cluster 1 (HIST1H1B, HIST1H1E, and HIST1H4C) and chromatin organization and transcriptional regulation (H2AFZ, HMGB2, and HMGN) were downregulated at late pseudotime stages. In contrast, clonotype 3 showed treatment-associated upregulation of genes involved in cell-cycle progression (UBE2C, CENPF, CENPM, and STMN1), proliferation markers (MKI67, TK1, and TYMS), and chromatin replication (HIST1H3B, HIST1H4C, H2AFZ, HMGB1, HMGB2, HMGN2, HIST1H1B, HIST1H1C, and HIST1H1E), indicating a highly proliferative state after treatment (**Supplementary Figures 9B–F**).

### Transcriptomic heterogeneity of tumor-associated macrophages within the same patient before and after therapeutic intervention

**Figure 5:**
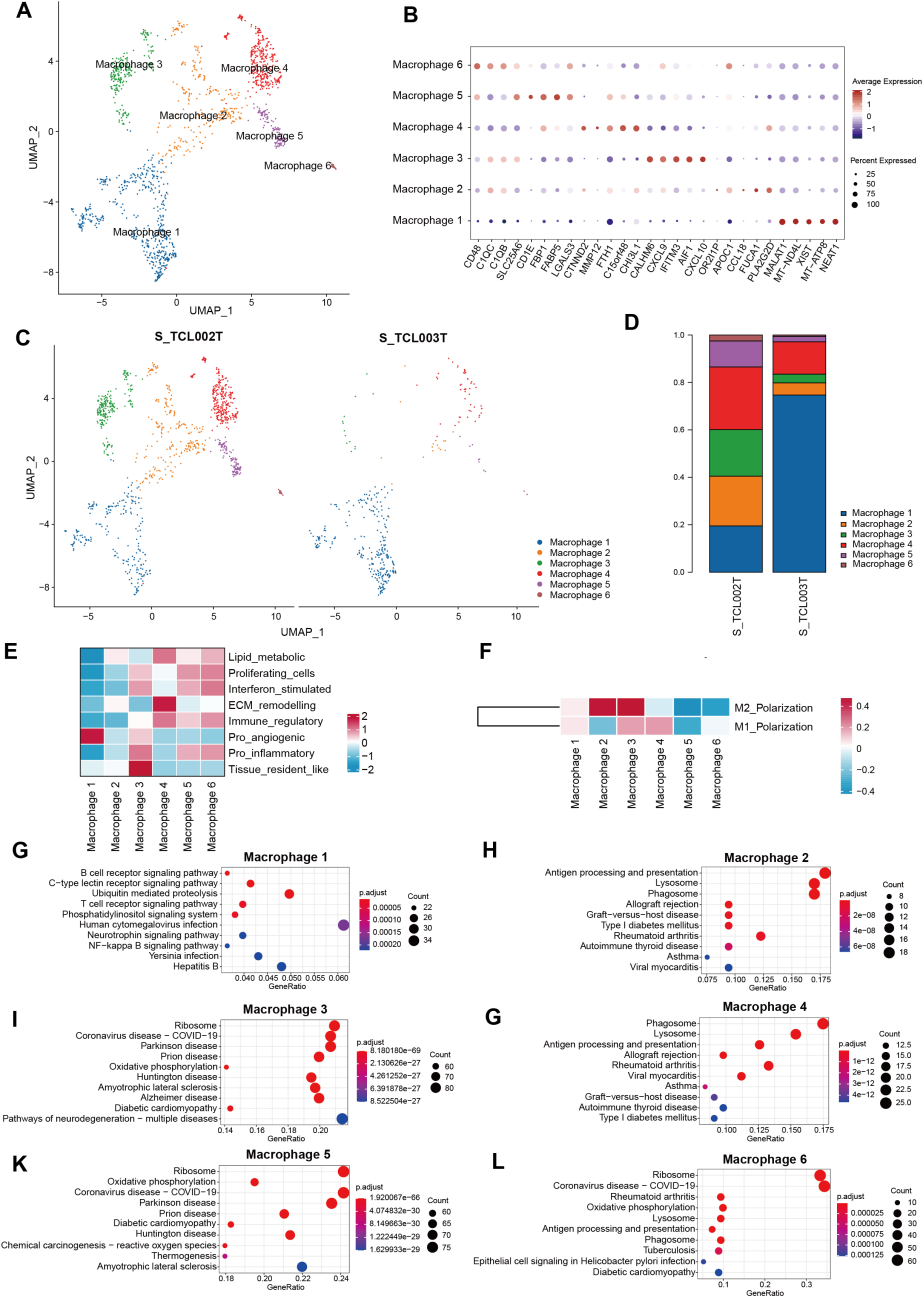
Transcriptomic heterogeneity of tumor-associated macrophages in paired samples from the same patient before and after dual epigenetic therapy. **(A)** UMAP visualization of macrophage populations (each dot represents an individual cell; distinct colors denote different cell subpopulations). **(B)** Dot plot depicting the mean expression levels of characteristic genes for six macrophage clusters (scaled proportionally). **(C)** UMAP visualizations of macrophages from paired samples collected from the same individual before and after treatment. **(D)** Histogram showing the relative proportions of macrophage subsets in paired samples before and after treatment. **(E)** Heatmap displaying functional enrichment scores for six macrophage clusters based on functionally related gene sets. **(F)** GSVA of M1 and M2 gene sets was performed on various macrophage subpopulations before and after treatment. **(G–L)** Bubble plots showing the results of the KEGG pathway enrichment analysis of upregulated differentially expressed genes in six macrophage clusters from samples before and after treatment.

Tumor-associated macrophages (TAMs) constitute the predominant innate immune population within the TME and are key contributors to the establishment of an immunosuppressive milieu, thereby facilitating tumor immune evasion (24). Accumulating evidence indicates that epigenetic reprogramming critically regulates the functional phenotypes of TAMs, including their recruitment, infiltration, and polarization toward an immunosuppressive state (25). To investigate the effect of epigenetic therapy on macrophage composition, we conducted a comparative analysis of macrophages in paired samples collected before treatment and after disease progression. We observed a significant reduction in the number of macrophages in the posttreatment progressive sample S_TCL003T relative to its pretreatment counterpart (**Supplementary Table 8**). Subsequent subclustering analysis based on signature gene expression profiles classified macrophages into six distinct clusters (**Figures 5A, B**). Notably, the proportions of macrophage 2–6 markedly decreased following treatment, whereas the frequency of macrophage 1 was significantly increased (**Figures 5C, D**). Functional gene set enrichment analysis further revealed that macrophage 1 exhibited a significantly higher enrichment score for pro-angiogenic functions (**Figure 5E**). In addition, GSVA of M1- and M2-polarization-related gene sets was conducted for each macrophage subpopulation (**Figure 5F**). This analysis demonstrated that macrophage 1 coexpressed molecular features associated with both M1 and M2 phenotypes, indicating a high degree of functional plasticity and the ability to dynamically shift between activation states in response to microenvironmental cues. In contrast, macrophage 2 and 3 displayed elevated M2 polarization scores, whereas macrophage 4 exhibited a strong M1 polarization signature. Macrophage 5 and 6 appeared to maintain an unpolarized or transitional phenotype. Furthermore, differential gene expression analysis across the macrophage clusters, followed by KEGG pathway enrichment analysis, revealed that macrophage 1 was significantly enriched in the B-cell receptor signaling pathway, T-cell receptor signaling pathway, and NF-κB signaling pathway (**Figures 5G–L**). These findings suggest a potential functional role for macrophage 1 in mediating resistance to dual epigenetic therapy.

**Figure 6:**
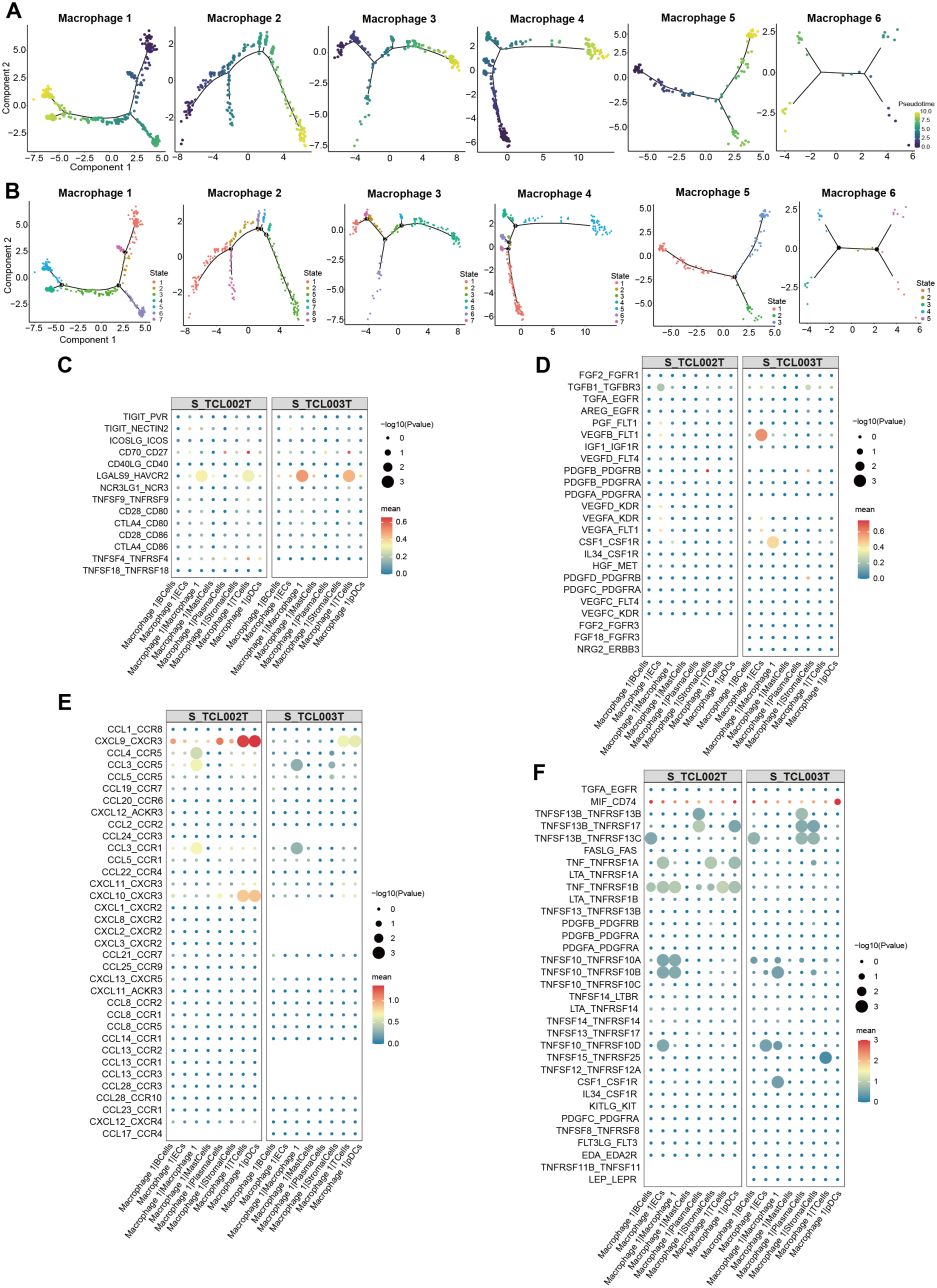
Pseudotime trajectory and cell–cell interaction analyses of tumor-associated macrophages in paired samples from the same patient before and after dual epigenetic therapy. **(A)** A single-cell analysis of the pseudotime trajectory was performed on six macrophage subtypes derived from the same patient before and after treatment using Monocle 2. A continuous value ranging from 0 to 10 was assigned to each cell as pseudotime. **(B)** The distribution of cells across distinct states along the pseudotime axis differs among each macrophage subtype. **(C)** Alterations in immune checkpoint-related ligand–receptor interactions between macrophage 1 and other immune cells in samples collected before and after treatment. **(D)** Growth factor-related ligand–receptor interactions. **(E)** Chemokine-associated ligand–receptor interactions. **(F)** Cytokine-associated ligand–receptor pairs.

Using Monocle 2, we performed pseudotime trajectory analysis to investigate dynamic changes in macrophage subpopulations before and after dual epigenetic therapy. In pretreatment samples, macrophage 2–6 were predominantly localized in the early phase of the pseudotime axis, whereas macrophage 1 was positioned in the late phase. In contrast, posttreatment samples showed selective enrichment of macrophage 1 in the early pseudotime stage (**Figures 6A, B**). We then analyzed genes whose expression shifted most significantly from the initiation to the termination of pseudotime to identify key transcriptional changes across differentiation trajectories. Macrophage 1 displayed high expression of immune response-related genes (HLA-DRA/DPA1/DRB5, CD74, C1QC, and LYZ) prior to treatment, with marked downregulation observed following therapy (**Supplementary Figure 10A**). In macrophage 2–6, genes associated with tumor-associated macrophage functions, including APOE, APOC1, and C1QA/B/C, and immune response pathways, including MMP9, CXCL9, CAPG, CTSB, and LGALS3, were also significantly downregulated after treatment (**Supplementary Figures 10B–F**).

To further characterize the effect of dual epigenetic therapy on the tumor immune microenvironment, we conducted differential cell–cell interaction analysis among key immune and stromal cell populations using paired samples collected before and after treatment. Among immune checkpoint-related ligand–receptor pairs, the LGALS9–HAVCR2 interaction between macrophage 1 and T cells was significantly strengthened after treatment (**Figure 6C**). In the context of growth factor signaling, the VEGFB–FLT1 axis exhibited enhanced crosstalk between macrophage 1 and ECs following therapy, while CSF1–CSF1R autocrine signaling within macrophage 1 was markedly upregulated (**Figure 6D**). Conversely, chemokine-mediated interactions, particularly those involving the CXCL9/10–CXCR3 axis, showed reduced connectivity between macrophage 1 and both T cells and pDCs after treatment (**Figure 6E**). Similarly, cytokine signaling via the TNF/LTA–TNFRSF1A/B pathway was attenuated between macrophage 1 and multiple interacting cell types in the posttreatment state (**Figure 6F**). Collectively, these shifts in ligand–receptor dynamics indicate a functional transition of macrophage 1 from an immune-activated state before therapy to an immunosuppressive state after treatment.

## Discussion

PTCL is a highly heterogeneous hematological malignancy, with approximately 70% of patients experiencing suboptimal responses to first-line therapy, and the emergence of tumor drug resistance frequently leads to R/R PTCL (26, 27). Despite recent advances in therapeutic strategies for PTCL, the characterization of the TME and transcriptional landscape in R/R PTCL remains limited and largely unexplored. In this study, we combined scTCR-seq with scRNA-seq, enabling high-throughput paired sequencing of TCRα and TCRβ chains at single-cell resolution (28, 29). This study represents a comprehensive single-cell characterization of TME heterogeneity, the T-cell clonal architecture, and transcriptional profiles in R/R PTCL. Furthermore, this study provides novel insights into the potential mechanisms underlying resistance to epigenetic therapies at single-cell resolution.

This study systematically characterized the transcriptional profile of dominant clonal populations across all samples, revealing the significant enrichment of oncogenic signaling pathways within these malignant clones. In the pretreatment cohort with R/R PTCL, dominant clonal T cells engaged in extensive communication with macrophages and B cells through multiple ligand–receptor interactions. Notably, immune checkpoint-related pairs—LGALS9–HAVCR2 and TIGIT–NECTIN2—demonstrated strong intercellular interactions across all the samples. Accumulating evidence indicates that LGALS9–HAVCR2 signaling is closely associated with tumorigenesis and immune evasion through the negative regulation of immune responses. Its coexpression with PD-1 on tumor-infiltrating lymphocytes suggests a synergistic mechanism underlying effector T-cell exhaustion and functional impairment (30–32). The TIGIT–NECTIN2 axis contributes to the modulation of an immunosuppressive microenvironment, with immune cell polarization toward immunosuppressive phenotypes reflecting a hallmark protumorigenic immune landscape (33). CXCL13–CXCR5 and CCL5–CCR5 exhibited robust signaling activity. The CXCL13–CXCR5 axis promotes the recruitment of regulatory B cells while inhibiting their migration into germinal centers, thereby impairing antigen-driven activation and maturation and fostering an immunosuppressive milieu (34). Elevated expression of CCL5 has been consistently linked to adverse clinical outcomes and supports tumor progression by functioning as a tumor growth factor, promoting angiogenesis, and facilitating immune escape (35). MIF binding to CD74 drives immune evasion and increases tumor proliferation (36–38) while also suppressing innate immune activation and dampening adaptive immunity via the inhibition of cytotoxic T lymphocytes (CTLs) (39). These ligand–receptor interactions represent potential biomarkers and key molecular drivers in the pathogenesis of R/R PTCL, highlighting promising targets for future precision therapeutic strategies.

In the dominant clonal T cells of the same patient following dual epigenetic drug treatment, the genes encoding histone H1 family members (HIST1H4C, HIST1H1E, and HIST1H1B), along with key chemokines (CXCL13, CCL4, CXCL9, and CCL3) and MHC class II genes (HLA-DRB5 and HLA-DQA2), were significantly downregulated. High CXCL13 expression is consistently associated with prolonged overall survival and improved responsiveness to immune checkpoint blockade (ICB) therapy (40). CXCL13 serves as a functional marker for identifying intratumoral precursor and terminally differentiated tumor-reactive CD8^+^ T cells, with the number of CXCL13^+^CD8^+^ T cells strongly correlated with favorable clinical outcomes following ICB treatment (41). Increased expression of tumor-specific MHC class II and components of its associated pathway is linked to a superior prognosis and heightened antitumor immunity, underscoring their roles in effective immune surveillance and tumor control (42, 43). Our findings demonstrate that posttreatment progressive samples exhibit an increased proliferative capacity and immune evasion potential. The downregulation of genes encoding histone H1, chemokine-related genes, and MHC class II genes is associated with therapeutic resistance, suggesting their potential as predictive biomarkers for the response to epigenetic therapies.

Our findings demonstrate that among T cells harboring identical TCR clonotypes in paired pre- and posttreatment samples, only clonotype 3 exhibited sensitivity to dual epigenetic regulatory drug therapy. Functional enrichment analysis of shared clonotypes revealed consistent activation of the JAK–STAT signaling pathway, particularly the IL6_JAK_STAT3_SIGNALING signature. In nasal natural killer/T-cell lymphoma, aberrant activation of the JAK–STAT pathway has been mechanistically linked to chidamide resistance via chromatin remodeling (44). In posttreatment samples, the expression of histone H1-associated genes in clonotype 3 decreased at later stages, whereas the expression of MALAT1 increased during the same period. Accumulating evidence indicates that MALAT1 is involved in disease progression and therapeutic resistance in diverse hematologic malignancies (45–49). Taken together, these data suggest that the downregulation of histone H1-associated genes and the concurrent upregulation of MALAT1 may contribute to disease progression and resistance to epigenetic therapies in PTCL.

Epigenetic regulation plays a pivotal role in modulating the interactions among cancer cells, immune cells, and stromal cells, as well as the crosstalk between malignant and immune compartments, thereby shaping the functional architecture of the tumor immune microenvironment (50). TAMs, the predominant immune cell population within this ecosystem, exhibit substantial phenotypic and functional heterogeneity, encompassing both antitumor and protumorigenic properties (51). In samples exhibiting disease progression following therapy, only macrophage 1 significantly expanded in frequency after treatment. Prior to treatment, the expression of antigen-presenting genes (HLA-DRA, DPA1, DRB5, CD74, C1QC, and LYZ) was high in macrophage 1. These genes were markedly downregulated after treatment, suggesting a loss of antigen-presenting capacity and functional impairment in this subset. An analysis of intercellular interactions in post-treatment samples revealed enhanced LGALS9–HAVCR2 ligand–receptor interactions between macrophage 1 and T cells. Signaling through the VEGFB–FLT1 and CSF1–CSF1R pathways—mediated between macrophage 1 and ECs or via autocrine loops within macrophage 1 itself—was also strengthened. In contrast, TNF/LTA–TNFRSF1A/B-mediated communication between macrophage 1 and various immune cell populations was diminished. The LGALS9–TIM3 pathway suppresses T-cell activity and promotes an immunosuppressive milieu (52–54). The CXCL10–CXCR3 axis enables tumor-associated neutrophils to impair cytotoxic T lymphocyte (CTL)-mediated antitumor immunity (55). Within the TME of R/R PTCL, macrophages may contribute to immune evasion by upregulating LGALS9 expression, leading to T-cell exhaustion. Collectively, therapy-induced alterations in intercellular communication—particularly increased LGALS9–HAVCR2 and CSF1–CSF1R interactions—may serve as promising biomarkers for predicting the response and resistance to dual epigenetic therapies in clinical practice. Nevertheless, this study has several limitations. In particular, the longitudinal analysis was based on paired pre- and posttreatment samples from one patient. Therefore, the observed treatment-associated changes in clonal dynamics, macrophage remodeling, and ligand–receptor interactions should be interpreted as exploratory and hypothesis-generating. Validation in larger independent cohorts with additional paired samples is required to confirm their biological and clinical relevance as potential biomarkers of response and resistance to dual epigenetic therapy.

Through integrated scRNA-seq and scTCR-seq analyses, we report the transcriptomic landscape of R/R PTCL, revealing profound tumor heterogeneity. The polyclonality of T cells and upregulation of immune escape pathways provide critical insights into disease biology and inform potential strategies for clinical intervention. Changes in gene expression and cell–cell interactions may serve as potential biomarkers for monitoring therapeutic response, suggesting that the combination of epigenetic agents with immune-directed therapies represents a rational and promising strategy for future research in R/R PTCL. In particular, CD5-targeted CAR-T cells, which have been reported to achieve an 85.7% response rate in relapsed/refractory CD5^+^ T-cell malignancies while overcoming the fratricide commonly associated with targeting T-cell malignancies, provide a concrete cellular immunotherapy approach that could be rationally combined with epigenetic modulation in this setting (56).

## Data Availability

The data presented in the study are deposited in the CNGB Sequence Archive of China National GeneBank DataBase, accession number CNP0009706. All other data generated or analyzed in this study are available from the corresponding authors upon reasonable request.
