# Supplementary material for: Single-cell sequencing uncovers clonal dynamics profiles and therapeutic resistance biomarkers in relapsed and refractory peripheral T-cell lymphoma
Source: Front Immunol. 2026 Jul 10;17:1790664. doi: 10.3389/fimmu.2026.1790664 (PMC13395935; doi:10.3389/fimmu.2026.1790664)
Supplement: Supplementary file 1 [file DataSheet1.docx]

**Supplementary table legends:**

**Supplementary Table S1.** Clinical characteristics and biopsy sampling details of patients undergoing puncture procedures

| Sample name | Sex | Age (years) | Pathologic diagnosis | Clinical stages | PIT score | Specific treatment at sampling | Group |
| --- | --- | --- | --- | --- | --- | --- | --- |
| S_TCL001T | F | 55 | PTCL-NOS | Ⅲ A | 1 | CHOPE | Pretreatment |
| S_TCL002T | F | 59 | PTCL-NOS | Ⅲ B | 0 | CHOPE |  |
| S_TCL004T | M | 65 | AITL | Ⅳ B | 2 | CHOPE |  |
| S_TCL006T | M | 59 | PTCL-NOS | Ⅳ B | 1 | CHOPE+ DICE |  |
| S_TCL008T | M | 63 | PTCL-NOS | Ⅲ A | 2 | CHOPE+ ICE |  |
| S_TCL009T | M | 70 | AITL | Ⅳ B | 2 | CHOP+ chidamide |  |
| S_TCL003T | F | 60 | PTCL-NOS | Ⅲ B | 0 | CHOPE | Progressive disease |

**Supplementary Table S2**. Quality control of single-cell transcriptome sequencing samples

| Sample name | Total Genes Detected | Mean Reads per Cell | Median Genes per Cell | Median UMI Counts per Cell | Valid Barcodes | Estimated Number of Cells |
| --- | --- | --- | --- | --- | --- | --- |
| S_TCL001T | 21265 | 35053 | 1068 | 2276 | 88.7% | 11282 |
| S_TCL002T | 21483 | 16934 | 1281 | 2494 | 87.5% | 19003 |
| S_TCL003T | 22541 | 27941 | 1547 | 3129 | 89.5% | 13512 |
| S_TCL004T | 22149 | 88604 | 1472 | 3355 | 90.0% | 4976 |
| S_TCL006T | 24055 | 32866 | 733 | 1446 | 88.1% | 12408 |
| S_TCL008T | 26237 | 76726 | 1950 | 4548 | 85.2% | 7574 |
| S_TCL009T | 24180 | 52945 | 839 | 1473 | 81.4% | 11209 |

**Supplementary Table S3.** Cell types and their corresponding typical markers

| Cell type | Typical marker |
| --- | --- |
| T cell | CD2, CD3D, TRAC, NKG7, CD69, CCL5, CD96 |
| B cell | CD79A, CD79B, MS4A1 |
| Plasma cell | CD79A, MZB1, JCHAIN, IGHG1 |
| Mononuclear phagocyte | LYZ, C1QA, CCR7, CD14, VCAN |
| Plasma cell dendritic cells | IL3RA, CLEC4C, LILRB4, TCF4, TCL1A |
| Mast cell | SAB1, TPSB2, CPA3 |
| Stromal cell | DCN, COL1A1, RGS5 |
| Endothelial cell | PECAM1, KDR, VWF, CDH5 |

**Supplementary Table S4.** Top 5 DEGs of dominant clone T cells in each sample

| Sample name | Clonotype | Top5 differential genes |
| --- | --- | --- |
| S_TCL001T | 1 | CST7，GZMA，GZMA，NKG7，TRBV5-6 |
| S_TCL002T | 1 | XCL2，CST7，GZMK，TRBV5-4，TRAV4 |
|  | 2 | CAV1，TRBV12-4，TRAV27，TRBV27，CXLC13 |
|  | 3 | S1004A，FOXP3，TRAV20，TRBV6-5，TNFRSF4 |
|  | 4 | CST7，GZMK，TRBV5-4，TRAV4，TRBV24-1 |
|  | 5 | CST7，GZMK，TRBV5-4，TRAV4，TRBV24-1 |
|  | 6 | S1004A，TNFRSF4，TRAV22，TRBV6-1，TRAV13-2 |
|  | 7 | S1004A，TRAV19，TRBV15，TNFRSF4，CCND2 |
|  | 8 | LAIR2，TRBV19，TRAV16，LAG3，CD7 |
|  | 9 | TRBV24-1，CST7，GZMK，TRBV5-4，TRAV6 |
|  | 10 | HIST1H1E，CST7，GZMK，TRAV4，XCL2 |
|  | 11 | TRAV26-2，TRBV11-2，S100A4，LAG3，TNFRSF4 |
|  | 12 | S1004A，CD7，TRBV3-1，LAG3，TRAV6 |
| S_TCL004T | 1 | CD40LG，TRAV2，IGFBP4，TRAV6，CXCL13 |
| S_TCL006T | 1 | IL7R，IFITM1，S100A4，TRAV3，TRBV6-5 |
| S_TCL008T | 1 | CCL5，CCL4，TRAV12-1，GZMK,NKG7 |
|  | 2 | CD8A，NKG7，TRBV11-3，TRAV16，CCL4 |
| S_TCL009T | 1 | GZMA，CST7，GZMK，TRBV10-2，TRAV21 |
| S_TCL003T | 1 | TRAV4，NKG7，GZMA，CCL5，CST7 |
|  | 2 | TRAV4，NKG7，GZMA，GZMK，TRBV24-1 |
|  | 3 | TRBV12-4，TRAV27，TRBV27，CXCL13，SERPINE2 |
|  | 4 | CCL5，CST7，GZMK，TRAV6，TRBV5-4 |
|  | 5 | NKG7，GZMA，CCL5，CST7，GZMK |
|  | 6 | TRBV5-4，LTB，TNFRSF4，FTH1，CD7 |
|  | 7 | TRAV4，LTB，TRBV6-2，TNFRSF4，CD7 |
|  | 8 | TRBV6-1，TRAV17，FTH1，LTB，LINC01229 |
|  | 9 | LTB，TRBV9，TRAV12-2，FTH1，CD7 |

**Supplementary Table S5.** List of TCR clones with a frequency ≥40 in each sample (* the cell proportion is the percentage of the number of T cells expressing the clone to the total number of T cells in the sample)

| Sample name | Clonotype | Specific clone type information | Cell quantity | Cell proportio* |
| --- | --- | --- | --- | --- |
| S_TCL001­T | 1 | TRAV24-None-TRAJ27_TRBV5-6-None-TRBJ2-1 | 106 | 4.06 |
| S_TCL002T | 1 | TRAV4-None-TRAJ5_TRBV5-4-None-TRBJ1-2 | 2626 | 24.59 |
|  | 2 | TRAV27-None-TRAJ42_TRBV27-None-TRBJ2-3 | 1265 | 11.85 |
|  | 3 | TRAV20-None-TRAJ42_TRBV6-5-None-TRBJ2-5 | 331 | 3.10 |
|  | 4 | TRAV6-None-TRAJ36_TRBV5-4-None-TRBJ1-2 | 292 | 2.73 |
|  | 5 | TRAV4-None-TRAJ5_TRBV24-1-None-TRBJ2-7 | 149 | 1.40 |
|  | 6 | TRAV13-2-None-TRAJ54_TRBV6-1-None-TRBJ1-6 | 135 | 1.26 |
|  | 7 | TRAV19-None-TRAJ17_TRBV15-None-TRBJ2-1 | 88 | 0.82 |
|  | 8 | TRAV16-None-TRAJ23_TRBV19-None-TRBJ2-7 | 86 | 0.81 |
|  | 9 | TRAV6-None-TRAJ36_TRBV24-1-None-TRBJ2-7 | 60 | 0.56 |
|  | 10 | TRAV4-None-TRAJ5_TRBV27-None-TRBJ2-3 | 53 | 0.50 |
|  | 11 | TRAV26-2-None-TRAJ28_TRBV11-2-None-TRBJ2-3 | 45 | 0.42 |
|  | 12 | TRAV6-None-TRAJ40_TRBV3-1-None-TRBJ1-2 | 44 | 0.41 |
| S_TCL004T | 1 | TRAV6-None-TRAJ8_TRBV7-2-None-TRBJ2-1 | 237 | 16.04 |
| S_TCL006T | 1 | TRAV3-None-TRAJ27_TRBV6-5-None-TRBJ2-7 | 47 | 0.70 |
| S_TCL008T | 1 | TRAV12-1-None-TRAJ28_TRBV11-3-None-TRBJ1-6 | 256 | 6.66 |
|  | 2 | TRAV16-None-TRAJ53_TRBV11-3-None-TRBJ2-2 | 43 | 1.12 |
| S_TCL009T | 1 | TRAV21-None-TRAJ15_TRBV10-2-None-TRBJ2-5 | 1645 | 22.28 |
| S_TCL003T | 1 | TRAV4-None-TRAJ5_TRBV5-4-None-TRBJ1-2 | 1808 | 15.15 |
|  | 2 | TRAV4-None-TRAJ5_TRBV24-1-None-TRBJ2-7 | 1412 | 11.83 |
|  | 3 | TRAV27-None-TRAJ42_TRBV27-None-TRBJ2-3 | 716 | 6.00 |
|  | 4 | TRAV6-None-TRAJ36_TRBV5-4-None-TRBJ1-2 | 190 | 1.59 |
|  | 5 | TRAV6-None-TRAJ36_TRBV24-1-None-TRBJ2-7 | 132 | 1.11 |
|  | 6 | TRAV8-3-None-TRAJ13_TRBV5-4-None-TRBJ1-2 | 112 | 0.94 |
|  | 7 | TRAV4-None-TRAJ38_TRBV6-2-None-TRBJ2-3 | 91 | 0.76 |
|  | 8 | TRAV17-None-TRAJ22_TRBV6-1-None-TRBJ1-6 | 57 | 0.48 |
|  | 9 | TRAV12-2-None-TRAJ4_TRBV9-None-TRBJ2-3 | 45 | 0.38 |

**Supplementary Table S6.** Cell types and their corresponding typical markers

| Cell type | Typical marker |
| --- | --- |
| Primary T cell | IL7R, CCR7, SELL |
| Effector T cell | GZMK, CCL5, NKG7 |
| Follicular helper T cell | CXCL13, TOX2, PDCD1 |
| Regulatory T cell | Foxp3, IL2RA, CTLA4 |
| Memory resident T cell | ZNF683, ITGA1, ITGAE |
| Depleting T cell | TIGIT, LAG3, CTLA4 |

**Supplementary Table S7.** The frequency and percentage of the same clonotype cells in the samples before and after treatment

| No. | Clone name | Before treatment | | After treatment | |
| --- | --- | --- | --- | --- | --- |
|  |  | Frequency | proportion | Frequency | proportion |
| 1 | TRAV4-None-TRAJ5_TRBV5-4-None-TRBJ1-2 | 2626 | 24.59% | 1808 | 15.15% |
| 2 | TRAV27-None-TRAJ42_TRBV27-None-TRBJ2-3 | 1265 | 11.84% | 716 | 6.00% |
| 3 | TRAV20-None-TRAJ42_TRBV6-5-None-TRBJ2-5 | 331 | 3.10% | 12 | 0.10% |
| 4 | TRAV4-None-TRAJ5_TRBV24-1-None-TRBJ2-7 | 149 | 1.40% | 1412 | 11.83% |
| 5 | TRAV6-None-TRAJ36_TRBV24-1-None-TRBJ2-7 | 60 | 0.56% | 132 | 1.11% |

**Supplementary Table S8.** The frequency of macrophages in each sample and the percentage of total macrophages in the sample

| name | Before treatment | | After treatment | |
| --- | --- | --- | --- | --- |
|  | Frequency | proportion | Frequency | proportion |
| Macrophage 1 | 212 | 19.56% | 263 | 74.72% |
| Macrophage 2 | 227 | 20.94% | 18 | 5.11% |
| Macrophage 3 | 213 | 19.65% | 13 | 3.69% |
| Macrophage 4 | 286 | 26.38% | 48 | 13.64% |
| Macrophage 5 | 119 | 10.98% | 8 | 2.27% |
| Macrophage 6 | 27 | 2.49% | 2 | 0.57% |
| Macrophages | 1084 |  | 352 |  |

**Supplementary figure legends:**

**Supplementary Figure 1.** Single-cell transcriptomic profiles of 7 samples. **(A)** The UMAP visualization illustrates the cellular expression profiles of 7 samples collected before and after epigenetic drug treatment. The pretreatment group comprised six samples (S_TCL001T, S_TCL002T, S_TCL004T, S_TCL006T, S_TCL008T, and S_TCL009T), whereas the progressive disease group included one sample (S_TCL003T). **(B)** UMAP visualization of seven samples, where each color corresponds to one sample. **(C)** Dot plots of canonical cell type markers stratified by cell type. **(D)** Histograms showing the proportion of each cellular subpopulation in the two groups before and after epigenetic drug treatment.

**Supplementary Figure 2.** Dot plot showing the expression of signature genes in dominant clonal T cells compared with matched non-dominant T cells from the same tumor. Dot color represents the average scaled expression level, and dot size indicates the proportion of cells expressing each gene. **(A)** Sample S_TCL001T. **(B)** Sample S_TCL002T. **(C)** Sample S_TCL003T. **(D)** Sample S_TCL004T. **(E)** Sample S_TCL006T. (**F**) Sample S_TCL008T. (**G**) Sample S_TCL009T.

**Supplementary Figure 3.** ssGSVA analysis of HALLMARK gene sets based on differentially expressed genes globally upregulated in dominant clonal T cells from pretreatment samples. The heatmap shows the relative enrichment scores of representative HALLMARK pathways across samples. **(A)** Sample S_TCL001T. **(B)** Sample S_TCL002T. **(C)** Sample S_TCL004T. **(D)** Sample S_TCL006T. **(E)** Sample S_TCL008T. **(F)** Sample S_TCL009T.

**Supplementary Figure** **4.** Cell–cell interaction analysis showing that dominant clonal T cells from six pretreatment samples interacted with macrophages and B cells through multiple immune checkpoint-associated ligand–receptor pairs. **(A)** Sample S_TCL001T. **(B)** Sample S_TCL002T. **(C)** Sample S_TCL004T. **(D)** Sample S_TCL006T. **(E)** Sample S_TCL008T. **(F)** Sample S_TCL009T.

**Supplementary Figure 5.** The dominant clonal T cells from the six samples from the pretreatment group that interacted with macrophages and B cells through multiple ligand–receptor pairs associated with chemokine signaling. **(A)** Sample S_TCL001T. **(B)** Sample S_TCL002T. **(C)** Sample S_TCL004T. **(D)** Sample S_TCL006T. **(E)** Sample S_TCL008T. **(F)** Sample S_TCL009T.

**Supplementary Figure 6.** The dominant clonal T cells from the six samples from the pretreatment group that interacted with macrophages and B cells through multiple ligand–receptor pairs associated with cytokine signaling pathways. **(A)** Sample S_TCL001T. **(B)** Sample S_TCL002T. **(C)** Sample S_TCL004T. **(D)** Sample S_TCL006T. **(E)** Sample S_TCL008T. **(F)** Sample S_TCL009T.

**Supplementary Figure 7.** Functional characteristics of the overall dominant clonotypes in the same patient before and after treatment. **(A)** UMAP visualization of functional T cell subsets. **(B)** Histogram illustrating the proportion of functional T-cell subsets in each sample. **(C)** Dot plot depicting the mean expression levels of characteristic genes for functional T cells (scaled proportionally).

**Supplementary Figure 8.** Analysis of differentially expressed genes in clonally matched T cells from the same patient before and after treatment.

**Supplementary Figure 9.** Dynamic gene expression patterns of the same dominant clonal T-cell population along pseudotime trajectories in paired pre- and posttreatment samples from the same patient. **(A)** Distribution of cells from each sample across distinct cell states along the pseudotime trajectory. **(B–F)** Dynamic changes in gene expression along the pseudotime trajectory in clonally identical T-cell populations before and after treatment. The x-axis represents pseudotime progression from early to late stages, while the y-axis lists individual genes. Each point corresponds to the mean expression level of a given gene at a specific pseudotime interval. Clusters denote groups of coexpressed genes identified through clustering analysis, where genes exhibiting similar temporal expression patterns are assigned to the same cluster.

**Supplementary Figure 10.** Dynamic gene expression trajectories of six tumor-associated macrophage subclusters across pseudotime before and after treatment. **(A)** Macrophage 1. **(B)** Macrophage 2. **(C)** Macrophage 3. **(D)** Macrophage 4. **(E)** Macrophage 5. **(F)** Macrophage 6.
