# Supplementary figures and images for: Single-cell sequencing uncovers clonal dynamics profiles and therapeutic resistance biomarkers in relapsed and refractory peripheral T-cell lymphoma
Source: Front Immunol. 2026 Jul 10;17:1790664. doi: 10.3389/fimmu.2026.1790664 (PMC13395935; doi:10.3389/fimmu.2026.1790664)

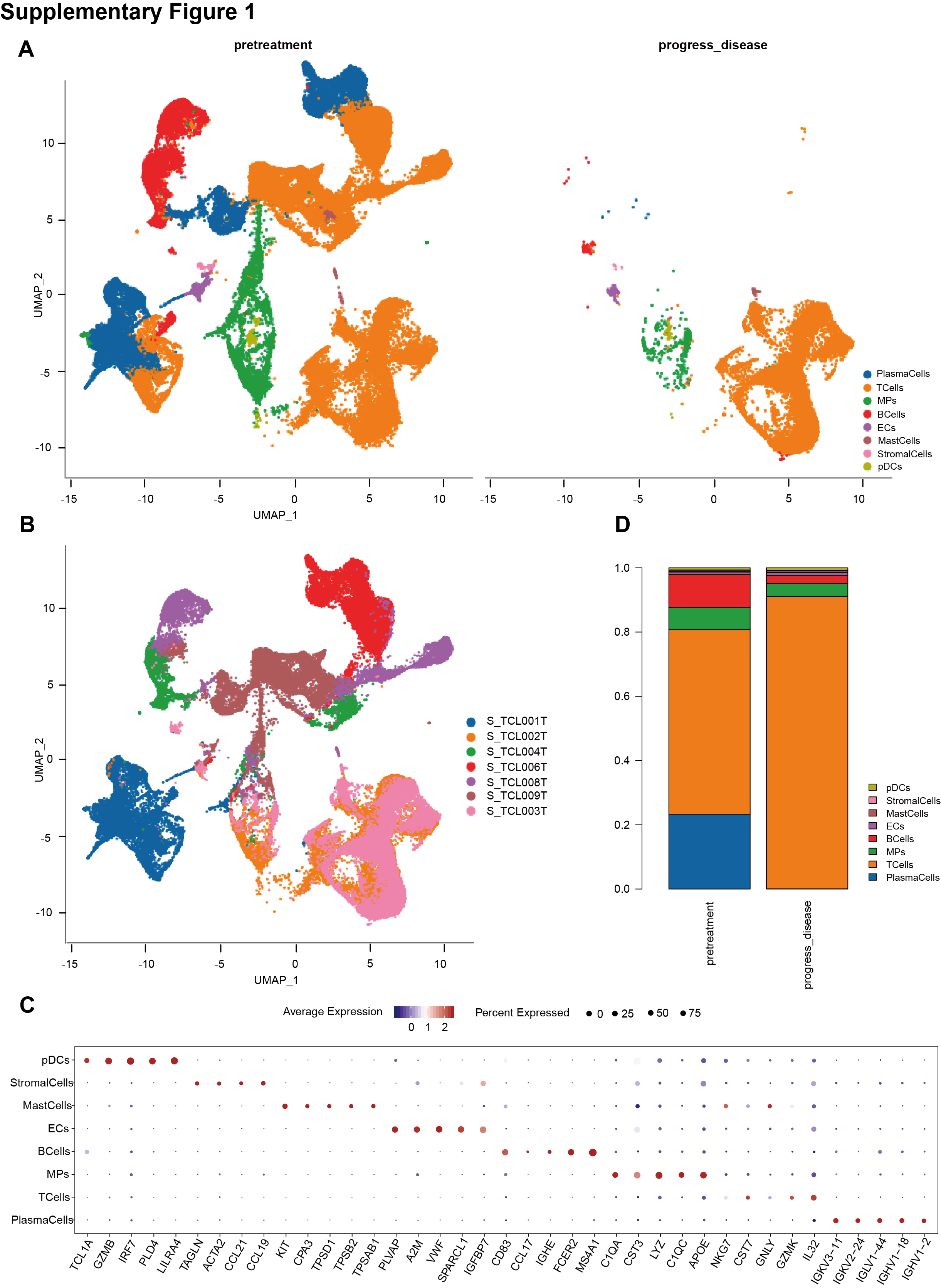

Supplement: Supplementary file 2 [file Image1.tif]

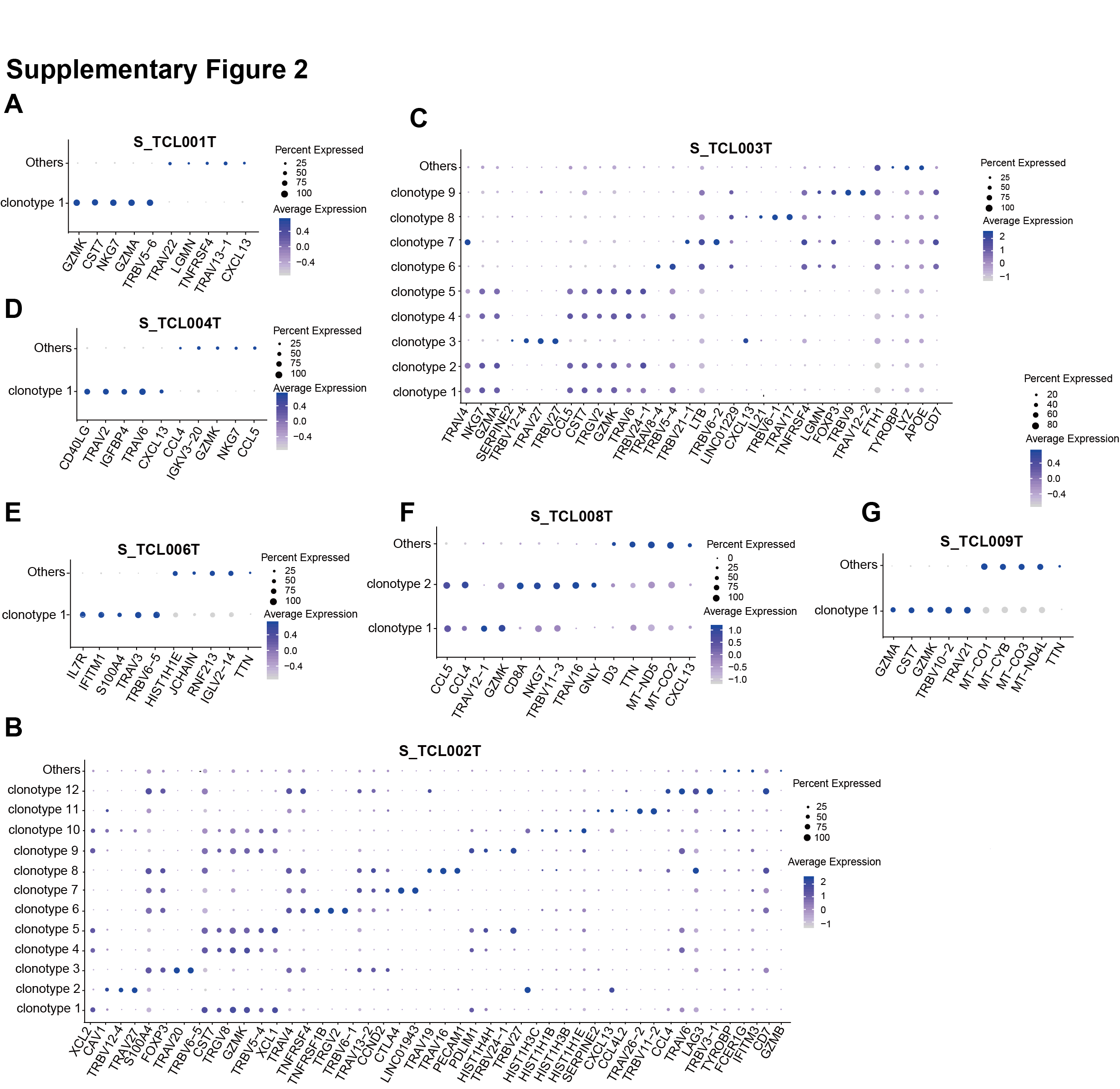

Supplement: Supplementary file 3 [file Image2.tif]

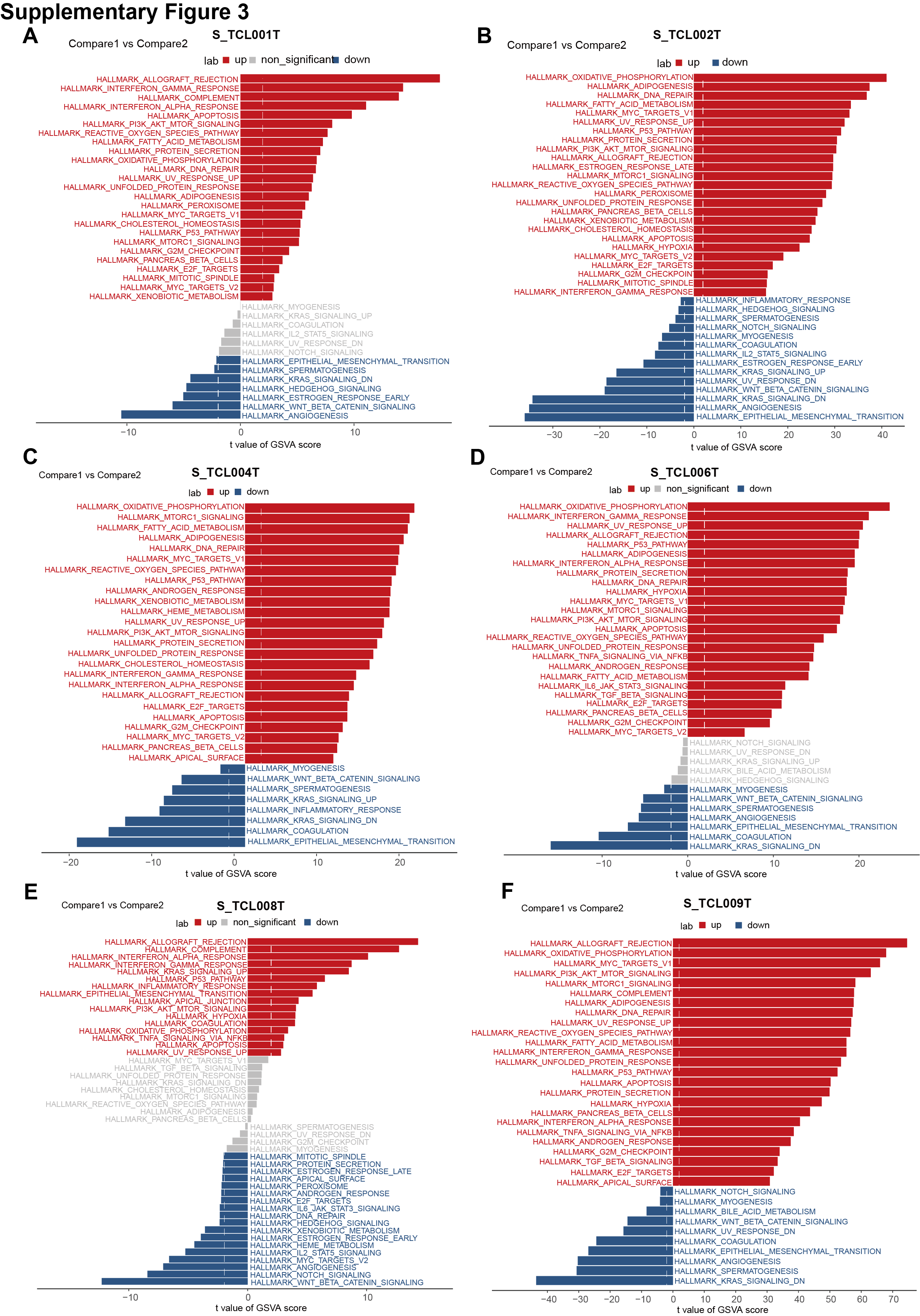

Supplement: Supplementary file 4 [file Image3.tif]

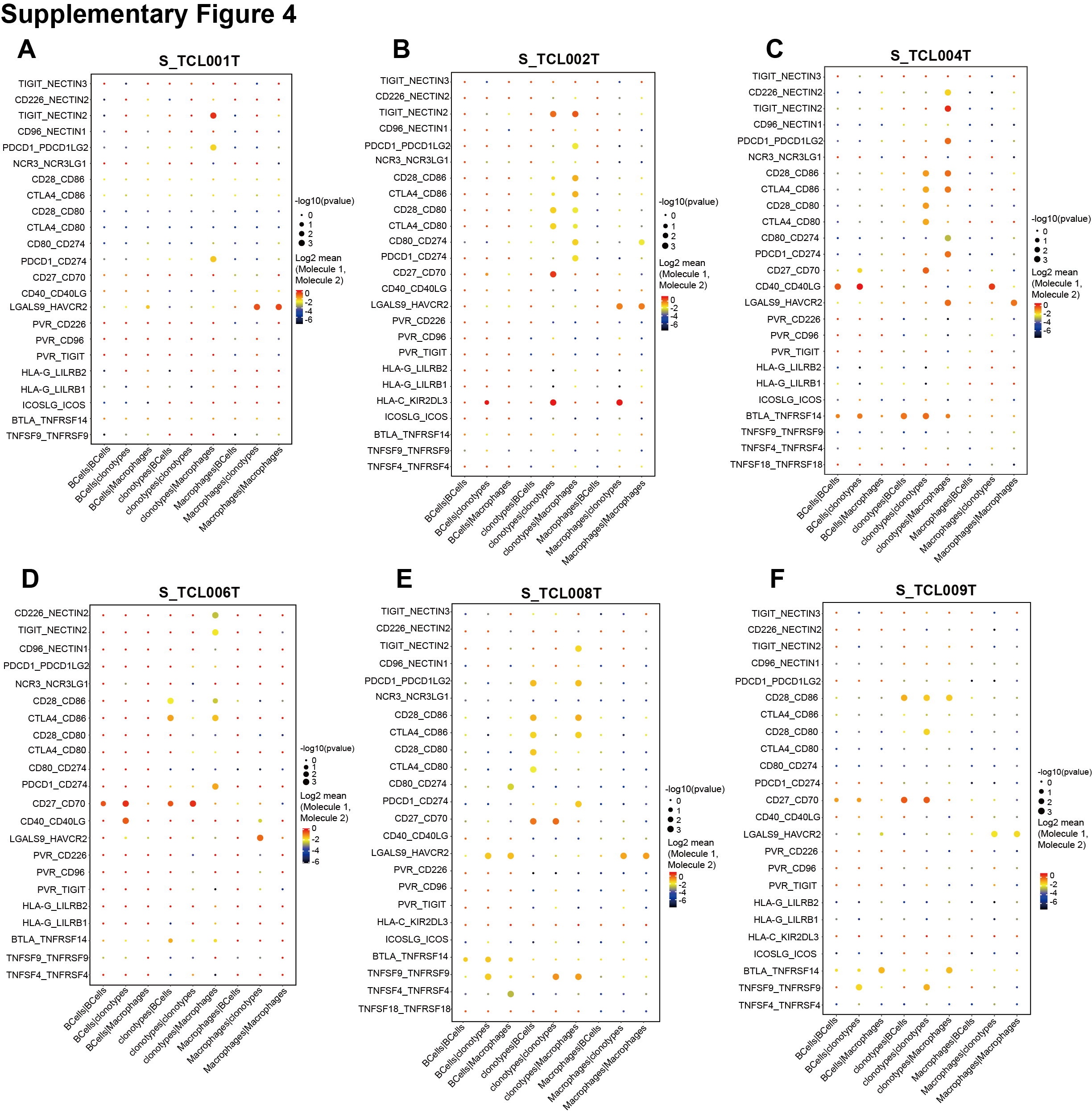

Supplement: Supplementary file 5 [file Image4.tif]

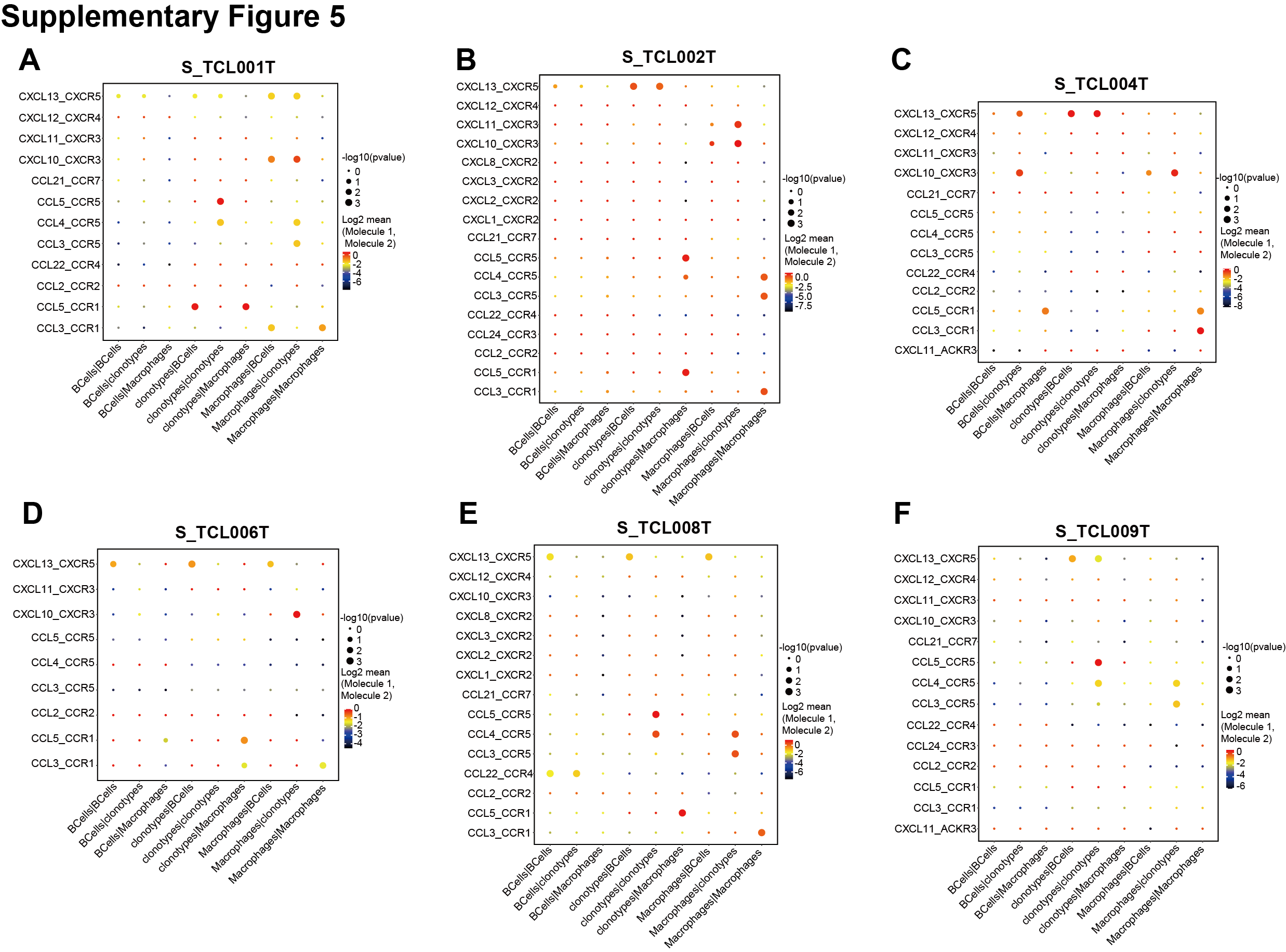

Supplement: Supplementary file 6 [file Image5.tif]

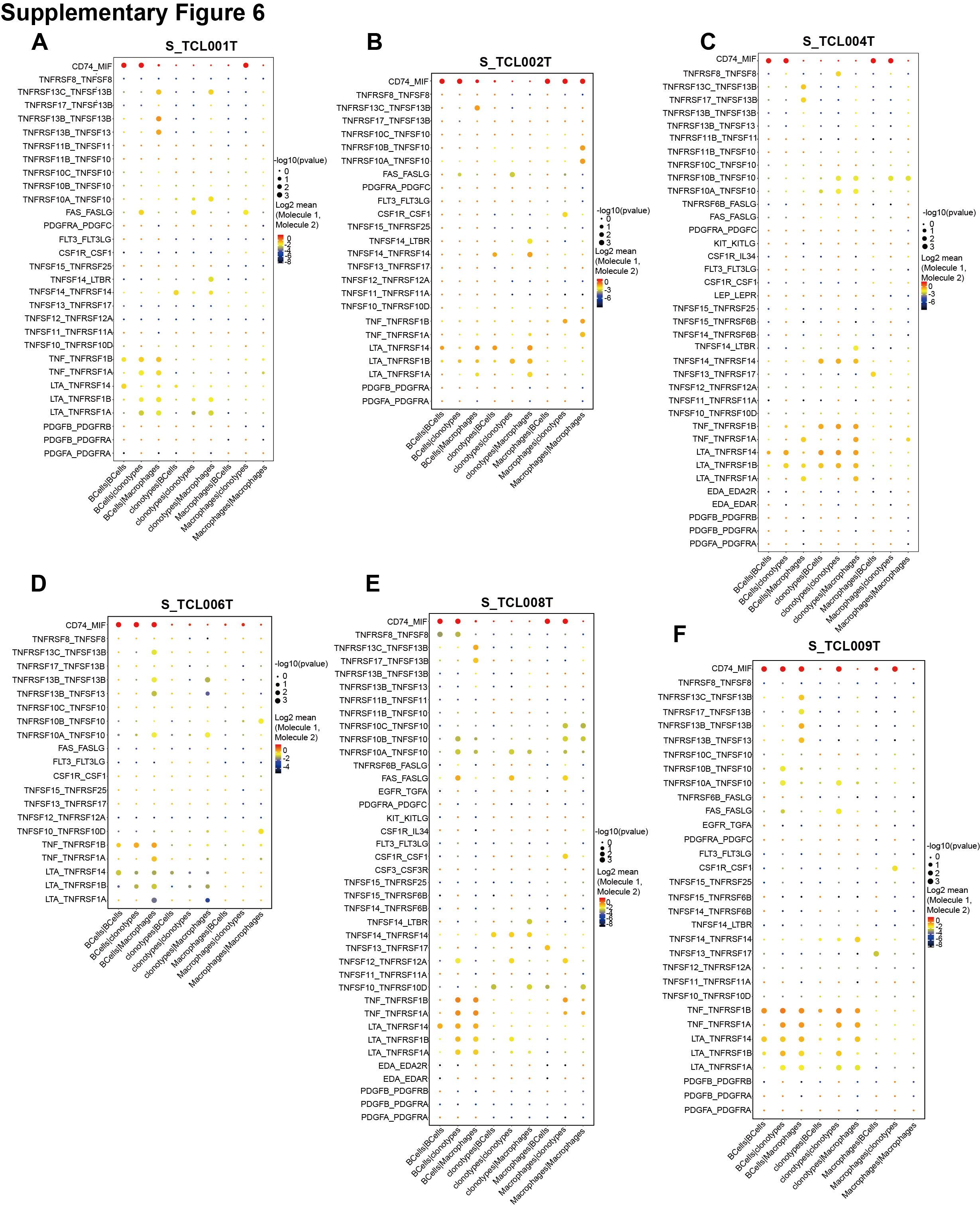

Supplement: Supplementary file 7 [file Image6.tif]

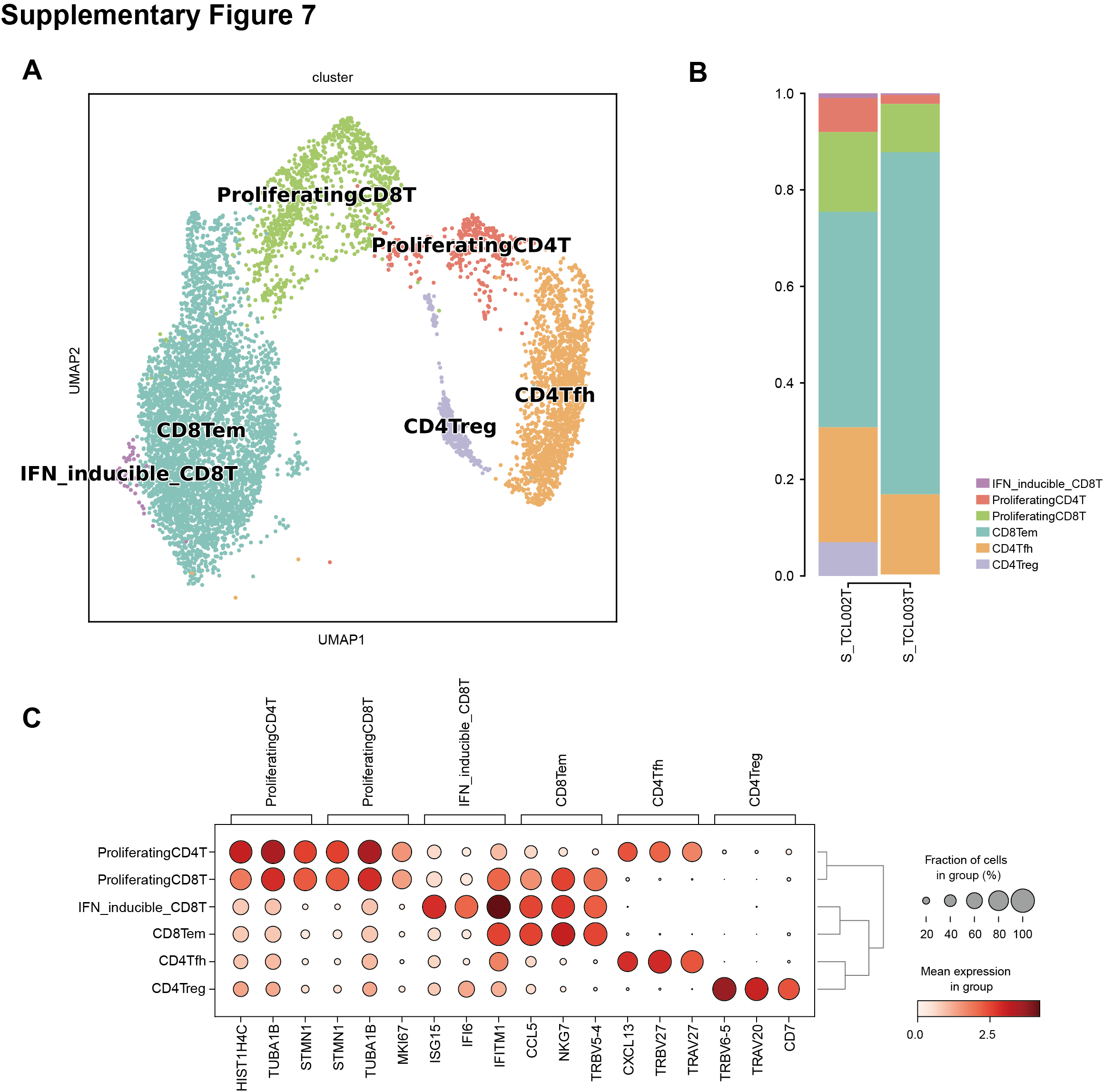

Supplement: Supplementary file 8 [file Image7.tif]

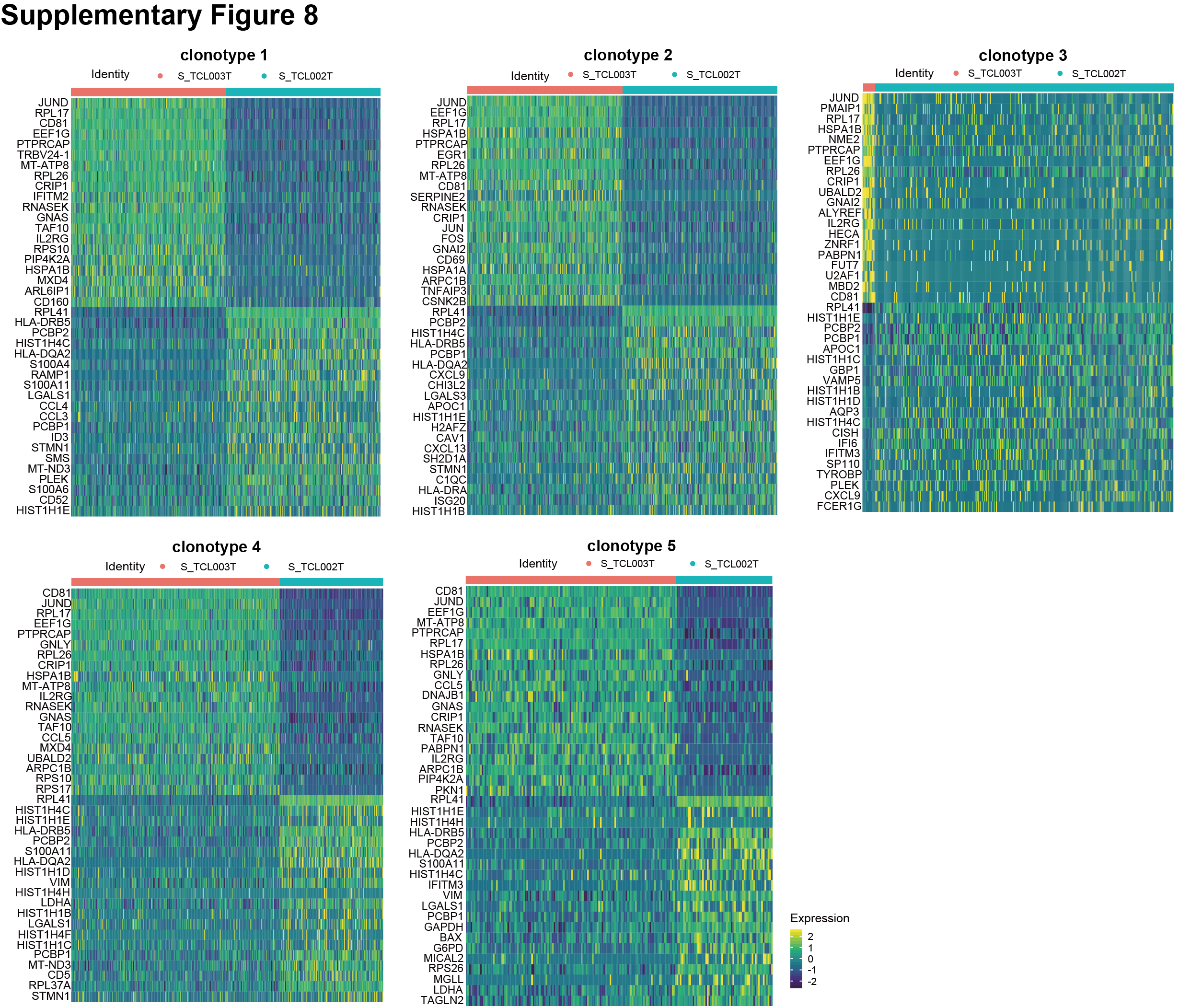

Supplement: Supplementary file 9 [file Image8.tif]

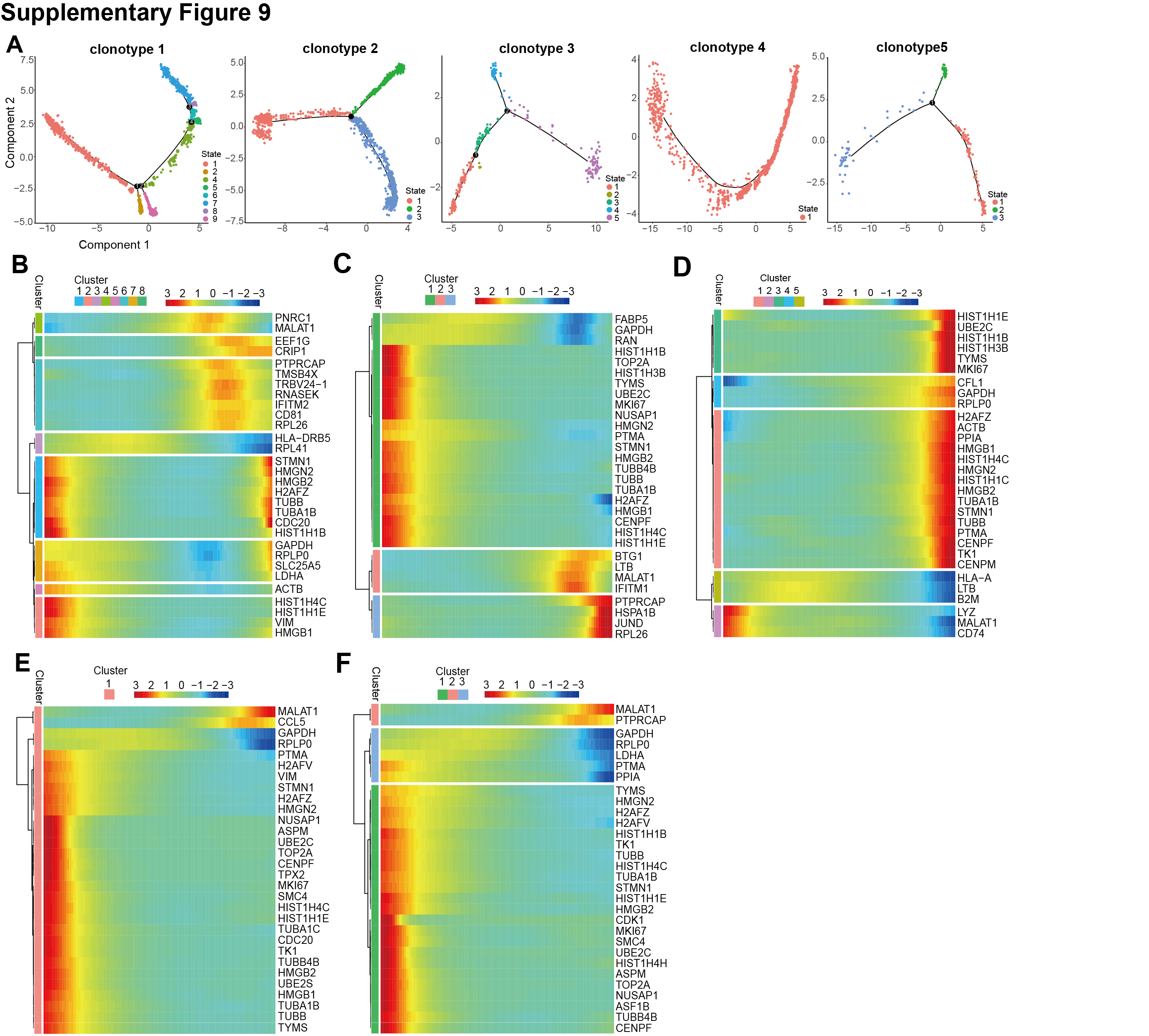

Supplement: Supplementary file 10 [file Image9.tif]

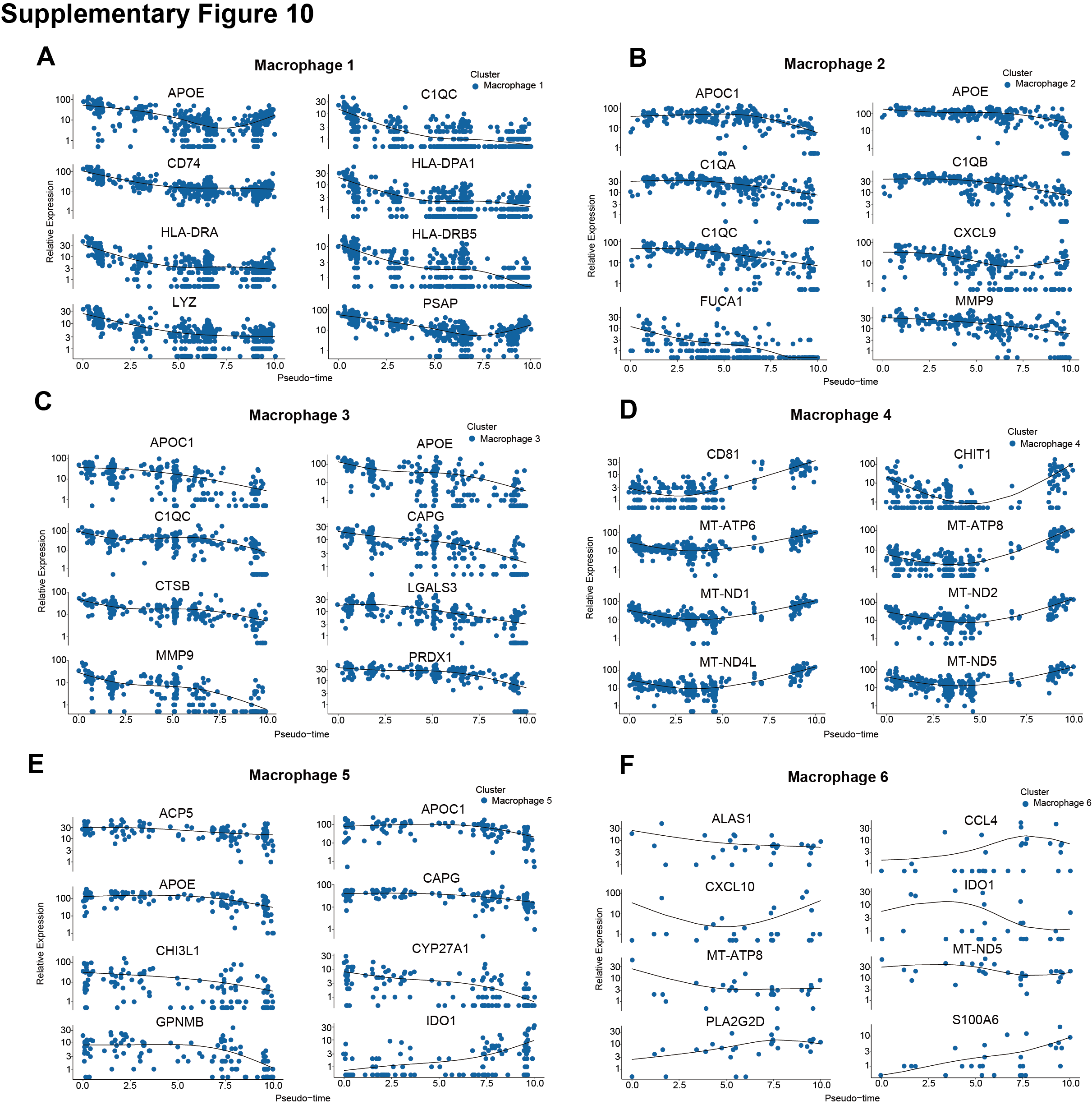

Supplement: Supplementary file 11 [file Image10.tif]
